# Modeling of the N-Glycosylated Transferrin Receptor Suggests How Transferrin Binding Can Occur within the Surface Coat of *Trypanosoma brucei*

**DOI:** 10.1371/journal.ppat.1002618

**Published:** 2012-04-05

**Authors:** Angela Mehlert, Mark R. Wormald, Michael A. J. Ferguson

**Affiliations:** 1 Division of Biological Chemistry and Drug Discovery, College of Life Sciences, University of Dundee, Dundee, United Kingdom; 2 Oxford Glycobiology Institute, Department of Biochemistry, University of Oxford, Oxford, United Kingdom; Washington University School of Medicine, United States of America

## Abstract

The transferrin receptor of bloodstream form *Trypanosoma brucei* is a heterodimer encoded by expression site associated genes 6 and 7. This low-abundance glycoprotein with a single glycosylphosphatidylinositol membrane anchor and eight potential N-glycosylation sites is located in the flagellar pocket. The receptor is essential for the parasite, providing its only source of iron by scavenging host transferrin from the bloodstream. Here, we demonstrate that both receptor subunits contain endoglycosidase H-sensitive and endoglycosidase H-resistant N-glycans. Lectin blotting of the purified receptor and structural analysis of the released N-glycans revealed oligomannose and paucimannose structures but, contrary to previous suggestions, no poly-N-acetyllactosamine structures were found. Overlay experiments suggest that the receptor can bind to other trypanosome glycoproteins, which may explain this discrepancy. Nevertheless, these data suggest that a current model, in which poly-N-acetyllactosamine glycans are directly involved in receptor-mediated endocytosis in bloodstream form *Trypanosoma brucei*, should be revised. Sequential endoglycosidase H and peptide-N-glycosidase F treatment, followed by tryptic peptide analysis, allowed the mapping of oligomannose and paucimannose structures to four of the receptor N-glycosylation sites. These results are discussed with respect to the current model for protein N-glycosylation in the parasite. Finally, the glycosylation data allowed the creation of a molecular model for the parasite transferrin receptor. This model, when placed in the context of a model for the dense variant surface glycoprotein coat in which it is embedded, suggests that receptor N-glycosylation may play an important role in providing sufficient space for the approach and binding of transferrin to the receptor, without significantly disrupting the continuity of the protective variant surface glycoprotein coat.

## Author Summary

The tsetse fly transmitted parasite that causes human African trypanosomiasis, or sleeping sickness, scavenges iron from the bloodstream of the infected individual so that it can live, multiply and ultimately cause disease. To do this, it places a glycoprotein (a protein with carbohydrate chains attached) called the transferrin receptor on its surface to capture circulating human transferrin, an iron transport protein. It then internalizes transferrin receptor/transferrin complex and digests the transferrin part, releasing the iron for its own use. By analyzing the parasite transferrin receptor, we have been able to describe the carbohydrate chains of the transferrin receptor and thus complete a molecular model of this important glycoprotein. We have further built models of how we expect this low abundance glycoprotein will sit in the surface coat of the parasite, which is made of millions of copies of another glycoprotein. The results provide a ‘molecule's eye view’ of how the carbohydrate chains of the transferrin receptor provide the space necessary for the transferrin to bind to it without disrupting the protective coat.

## Introduction

The tsetse-transmitted *Trypanosoma brucei* group of parasites cause human African trypanosomiasis and nagana in cattle and constitute a serious health problem for people and livestock in 36 countries of sub-Saharan Africa. *T. brucei* exists in the mammalian host as the bloodstream form trypomastigote and in the midgut of the tsetse fly vector as the procyclic form. The major surface molecules of the bloodstream form parasite are the glycosylphosphatidylinositol (GPI) anchored [1]–[4] and N-glycosylated [3]–[6] variant surface glycoproteins (VSGs), 5×10^6^ homodimers of which form a dense monolayer over the whole trypanosome [4]. The ability of individual trypanosomes to switch expression from one VSG gene to another gives rise to antigenic variation by which the parasite population survives the host acquired immune response [7]. Other less abundant glycoproteins are arranged either apparently randomly within the VSG coat, like the invariant glycoproteins ISG65 and ISG75 [8], [9], while others have specific surface locations, like Fla1 which locates to the flagellar adhesion zone [10] and the transferrin receptor which locates to the flagellar pocket [11]. Still other glycoproteins are located primarily in intracellular sites, like lysosomal p67 [12], Golgi and lysosomal tGLP1 [13], endoplasmic reticulum GPIdeAc [14] and endosomal TbMBAP1 [15]. The surface of the procyclic form parasite is dominated by 3×10^6^ copies of the GPI-anchored and N-glycosylated procyclin glycoproteins [4], [16], [17], about 1×10^6^ free GPI glycolipids [18], [19] and a high-molecular weight glycoconjugate complex [20], [21]. While this life cycle stage shares some glycoproteins with the bloodstream form, like p67, tGLP1 and Fla1, others are clearly bloodstream form specific, like ISG65, ISG75, TbBMAP1 and the expression site associated gene (ESAG) 6 and ESAG7 subunits of the heterodimeric *T. brucei* transferrin receptor (TfR).

Some of these glycoproteins are encoded by polygene families, causing sequence heterogeneity in the populations expressed by the trypanosomes. In the case of the TfR ESAG6/ESAG7 subunits, the *ESAG6* and E*SAG7* genes are associated with telomeric VSG expression sites such that one dominant ESAG6/ESAG7 pair dominates according to which site (and VSG variant) is being expressed [22]. However, there is also some transcriptional breakthrough from other expression sites, as the *ESAG6* and *ESAG7* genes are immediately adjacent to the expression site promoters, providing some sequence heterogeneity in all TfR preparations [23]. There is functional significance with respect to which ESAG6/ESAG7 pair is expressed due to their different affinities for transferrins from different mammalian species [24], [25]. While there are quite complete data on the GPI anchor and N-glycan structures and N-glycosylation site occupancies of specific VSGs and procyclins [1]–[6], [17], [26] and on the structures of the total N-glycan repertoires of the bloodstream form [27], [28] and procyclic form [29], [30] of the parasite, there is a paucity of data of the glycosylation status of other specific *T. brucei* glycoproteins. In this paper, we describe the N-glycosylation status of the ESAG6 and ESAG7 subunits of the transferrin receptor (TfR) and, together with our previous description of the GPI anchor of the ESAG6 subunit [31], provide a relatively complete description of the glycosylation status of this low abundance (approximately 3000 copies per cell [32]) but nutritionally essential [33] glycoprotein. The results are discussed in the context of proposed mechanisms of protein N-glycosylation [34]–[37] and endocytosis [38] in *T. brucei*. We also build a molecular model of the glycosylated ESAG6/ESAG7 transferrin receptor, surrounded by models of glycosylated VSG molecules, to visualize how this receptor sits in the VSG coat on the flagellar pocket membrane and how it might bind its transferrin ligand.

## Results

### Purification and endoglycosidase digestion of *T. brucei* TfR

**Figure 1:**
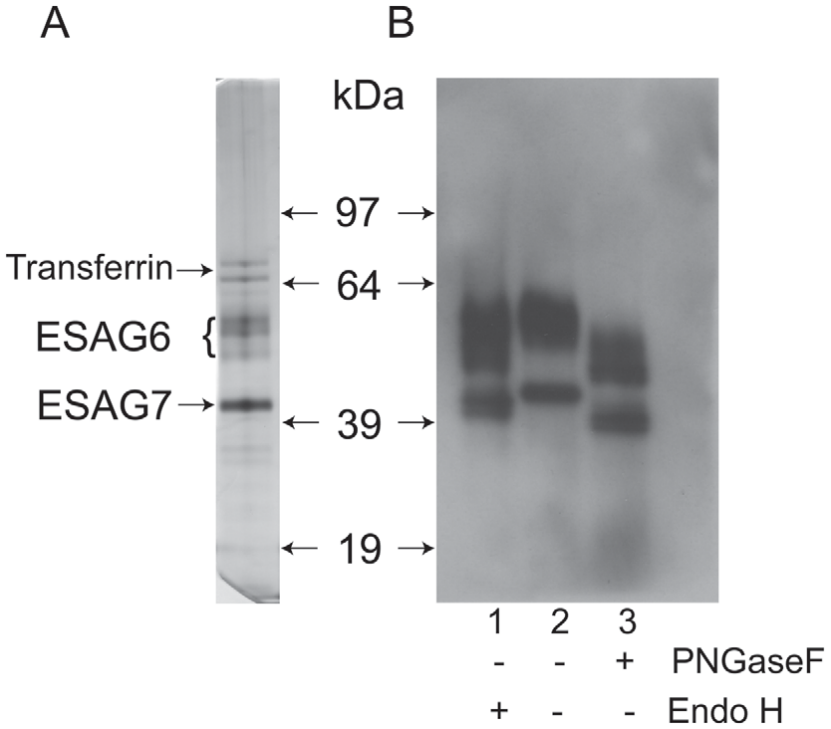
SDS-PAGE analysis of purified *T. brucei* TfR and endoglycosidase digestions. (A) An aliquot of *T. brucei* TfR, affinity purified on transferrin-Sepharose, was analyzed by SDS-PAGE and silver staining. (B) Aliquots of purified TfR were incubated with Endo H (lane 1), PNGase F (lane 3) or mock treated (lane 2) and analyzed by SDS-PAGE and Western blot with an antibody that reacts with the ESAG6 and ESAG7 subunits of TfR.

The transferrin receptor (TfR) was purified by affinity chromatography on immobilized transferrin, following the method first described by Steverding and Overath [39]. An aliquot was analyzed by SDS-PAGE and silver staining, which showed the characteristic ESAG6 and ESAG7 subunits (Figure 1A). The identities of the ESAG6 and ESAG7 components were confirmed by excision of the individual bands, in gel tryptic digestion and proteomic analysis (data not shown). Endoglycosidase digests confirmed that both ESAG6 and ESAG7 carry N-linked oligosaccharides. Thus, digestion with both peptide N-glycosidase F (PNGase F), an enzyme that cleaves essentially all types of N-linked glycan, and Endoglycosidase H (Endo H), an enzyme that cleaves only oligomannose-type N-glycans, reduced the apparent molecular weights of both proteins, as judged by SDS-PAGE and Western blotting with anti-TfR antibodies (Figure 1B). However, PNGase F reduced the apparent molecular weights of both proteins more than Endo H (Figure 1B, compare lanes 1 and 3), suggesting that both proteins contain a mixture of Endo H-sensitive (i.e., oligomannose) and Endo H-resistant (*i.e.*, paucimannose and/or complex) N-glycans. The heterogeneity still apparent in ESAG6 following complete de-N-glycosylation with PNGase F is presumably due to the reported heterogeneity in the α-galactose side chains of the GPI anchor attached to this TfR subunit [31].

### Lectin blotting of *T. brucei* TfR

**Figure 2:**
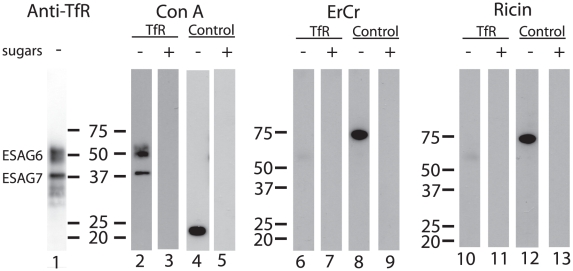
Lectin blots of purified TfR. Aliquots of purified *T. brucei* TfR (lanes 1, 2, 3, 6, 7, 10 and 11) and of bovine ribonuclease B, a positive control for ConA blotting (lanes 4 and 5), and bovine asialotransferrin, a positive control for ErCr lectin and ricin blotting (lanes 8, 9, 12 and 13), were separated by SDS-PAGE, transferred to nitrocellulose and subjected to blotting with anti-TfR antibody (lane 1), ConA (lanes 2–5), ErCr lectin (lanes 6–9) or ricin (lanes 10–13) in the absence (−) or presence (+) of the competing sugars α-methyl-mannose (lanes 3 and 5), lactose (lanes 7 and 9) or galactose and lactose (lanes 11 and 13). The positions of molecular weight markers are indicated for each group of blots.

Aliquots of purified TfR were separated by SDS-PAGE, blotted onto nitrocellulose and probed with anti-TfR antibody (Figure 2, lane 1) and by lectins. Consistent with the presence of oligomannose N-glycans, both ESAG6 and ESAG7 subunits gave a positive reaction with concanavalin A (ConA) (Figure 2, lane 2), as did the bovine ribonuclease B positive control glycoprotein (Figure 2, lane 4). These reactions were abolished when α-methyl-mannose was included in the blotting buffer (Figure 2, lanes 3 and 5), demonstrating the carbohydrate specificity of the ConA blots. However, neither of the ESAG subunits gave a significant reaction with the poly-LacNAc-specific tomato lectin (Figure S1) or, more importantly, with the far more permissive [40] N-acetyllactosamine (LacNAc) specific lectin from *Erythrina cristigalli* (ErCr) (Figure 2, lane 6) or with the terminal β-galactose-specific lectin ricin (Figure 2, lane 10). These experiments were performed under conditions where a strong reaction was seen against the positive control glycoprotein bovine asialotransferrin (Figure 2, lanes 8 and 12) and where the reactions with the ErCr and ricin lectins against the positive control were abolished by the inclusion of lactose or galactose plus lactose, respectively, in the blotting buffer (Figure 2, lanes 9 and 13). These data suggest that the Endo H-resistant N-glycans of ESAG6 and ESAG7 are not of the poly-LacNAc-containing complex type nor, indeed, even of the LacNAc-containing complex type and are, therefore, most likely of the paucimannose type.

### Release, radiolabeling and analysis of *T. brucei* TfR N-glycans

**Figure 3:**
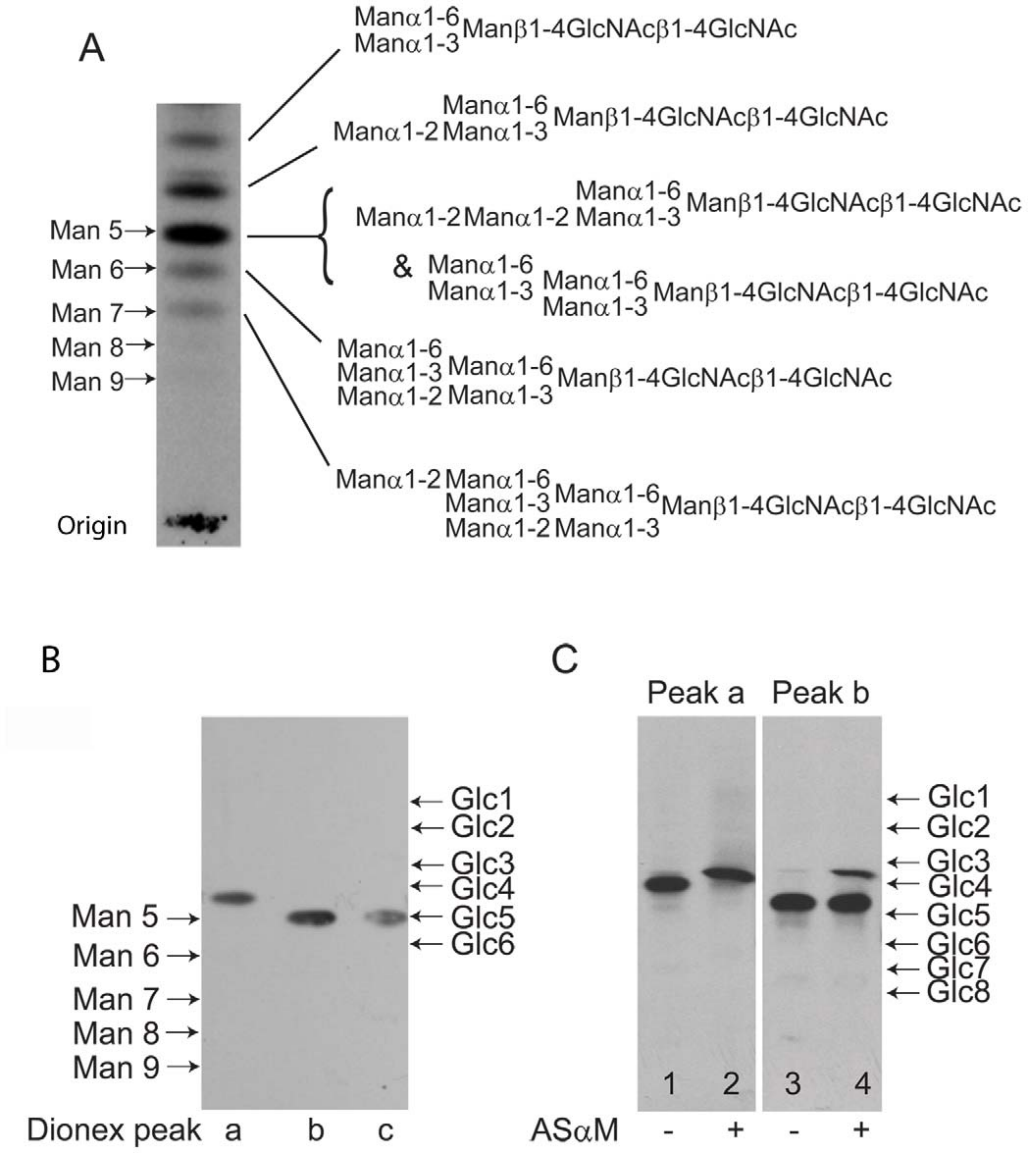
Fluorographs of HPTLC analyses of released and radiolabeled N-glycans from purified TfR. (A) Total N-glycan fraction of TfR released by PNGase F and radiolabeled by reduction with NaB[^3^H]_4_. The positions of oligomannose N-linked glycan standards reduced with NaB[^3^H]_4_ are shown on the left. The proposed structures of the principal TfR glycans are shown on the right. The top three biantennary structures (Man_3_GlcNAc_2_ to Man_5_GlcNAc_2_) are of the paucimannose series and the bottom three triantennary structures (Man_5_GlcNAc_2_ to Man_7_GlcNAc_2_) are of the oligomannose series. (B) The three major components of the radiolabeled TfR N-glycan fraction isolated by Dionex HPAEC (*peaks a, b and c*; lanes 1, 2 and 3, respectively). The positions of NaB[^3^H]_4_ reduced oligomannose N-linked glycan and dextran oligomer standards are shown on the left and right, respectively. (C) The paucimannose Man_4_GlcNAc_2_ structure (Dionex *peak a*) before (lane 1) and after (lane 2) digestion with Manα1-2Man specific α-mannosidase (ASαM) and the triantennary oligomannose Man_5_GlcNAc_2_structure Dionex *peak b* before (lane 3) and after (lane 4) digestion with ASαM. The positions of NaB[^3^H]_4_-reduced dextran oligomers are shown on right.

The lectin blotting experiments, described above, suggested that ESAG6 and ESAG7 contain oligomannose and paucimannose N-glycans. However, there remained the formal possibility that the Endo H-resistant N-glycan fraction might include complex N-glycans fully capped with terminal α-Gal residues, which could abrogate ricin and ErCr lectin binding to the sub-terminal β-Gal residues and LacNAc units, respectively, and for which there is precedent in some VSG N-linked glycans [5]. Therefore, to analyze the N-glycan structures further, total N-glycans were released from TfR with PNGase F, radiolabeled by reduction with NaB[^3^H]_4_ and analyzed by high-performance thin layer chromatography (HPTLC) alongside radiolabeled N-glycan standards (Figure 3A). A ladder of bands was observed, stretching from the position of Man_9_GlcNAc_2_ to Man_5_GlcNAc_2_, with two additional bands of higher Rf, possibly corresponding to Man_4_GlcNAc_2_ and Man_3_GlcNAc_2_ paucimannose species. Significantly, there were no bands with Rf values consistent with complex N-glycans capped with terminal α-Gal residues or with poly-LacNAc-containing N-glycans, like those found in VSG variant MITat1.7 [5] (Figure S2). The radioactive material at the origin of the TLC plate in (Figure 3A) is present in all NaB[^3^H]_4_-labeled samples, including commercial glycan standards (Figure S2). A sample of the mixture of labeled N-glycans was separated by Dionex high-pH anion exchange chromatography (HPAEC) and three major radioactive peaks were recovered (Figure S3). These were individually analyzed by HPTLC alongside authentic radiolabeled N-glycan standards and it was found that *peak b* and *peak c* co-migrated with Man_5_GlcNAc_2_ by HPTLC, while *peak a* migrated ahead of Man_5_GlcNAc_2_ and was assigned as a putative Man_4_GlcNAc_2_ structure (Figure 3B). Consistent with the latter assignment, digestion of the *peak a* material with the *Aspergillus saitoi* Manα1-2Man-specific α-mannosidase (ASαM) caused an increase in Rf equivalent to the removal of a single hexose (Figure 3C, compare lanes 1 and 2). In contrast, the majority of the material in the *peak b* fraction was resistant to ASαM (Figure 3C, compare lanes 3 and 4), suggesting that this is a tri-antennary Man_5_GlcNAc_2_ structure of the conventional oligomannose series. By inference, we assign the *peak c* material as the bi-antennary Man_5_GlcNAc_2_ structure of the paucimannose series and, indeed, a small component of the *peak b* material does digest with ASαM to lose two hexose residues, suggesting this is a small amount of bi-antennary Man_5_GlcNAc_2_ contamination from the adjacent *peak c* (Figure 3C, lane 4). Unfortunately, there was insufficient radiolabeled purified *peak c* material on which to perform a separate ASαM digest. The proposed structures of the main N-glycan species are shown in (Figure 3A). These structures are consistent with the data in (Figure 3A–C) and also draw on our prior knowledge of the structures of the oligomannose and paucimannose series in bloodstream form *T. brucei* [5], [6], [28].

### Analysis of TfR N-glycosylation site occupancy by LC-MS/MS

**Figure 4:**
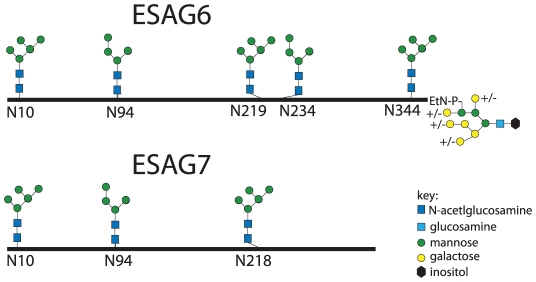
Glycosylation patterns of ESAG6 and ESAG7. Maps of the major N-glycan types at each N-glycosylation site along the polypeptide backbones of ESAG6 and ESAG7, based on the data in Figure 3 and Table 1, and the GPI glycan structure of ESAG6, based on [31].

The aforementioned endoglycosidase digestion results, lectin blots and N-glycan structural analyses strongly suggest that ESAG6 and ESAG7 contain both oligomannose and paucimannose N-glycans, but not complex N-glycans. Previous work has shown that bloodstream form *T. brucei* expresses two classes of oligosaccharyltransferase (OST] activity [34]–[37]: One that transfers Man_5_GlcNAc_2_ from Man_5_GlcNAc_2_-PP-Dol to N-glycosylation sequons in relatively acidic environments and another that transfers Man_9_GlcNAc_2_ from Man_9_GlcNAc_2_-PP-Dol to the remaining N-glycosylation sequons. These activities are encoded by the *TbSTT3A* and *TbSTT3B* genes, respectively [34]. We therefore subjected purified TfR to Endo H digestion followed by PNGase F digestion, resolved the double-digested ESAG6 and ESAG7 by SDS-PAGE and performed in-gel tryptic digestion and analyzed the resultant peptides by LC-MS/MS. Using this protocol [34], peptides encompassing Endo H-sensitive (oligomannose) N-glycosylation sites appear with a 203 Da shift, from the single GlcNAc residue left attached to the Asn residue by Endo H, and peptides encompassing Endo H-resistant (paucimannose) N-glycosylation sites appear with a 1 Da shift, from the conversion of Asn to Asp by PNGase F. Using this technique, we were able to positively identify three of the five N-glycosylation sites of ESAG6 as occupied, one (Asn94) with Endo H-resistant paucimannose N-glycans and two (Asn10 and Asn344) with Endo H-sensitive oligomannose N-glycans. The pI values of these Asn-Xaa-Ser/Thr sequons ±5 amino acid residues are consistent with their modification by TbSTT3A and TbSTT3B OST activities, respectively [34] (Table 1). Peptides encompassing the remaining two putative N-glycosylation sites, at Asn219 and Asn234, were not observed but their surrounding sequences would suggest that they are both modified by TbSTT3B OST and are likely to carry oligomannose structures (Table 1). In the comparable ESAG7 analysis, we positively identify one (Asn10) of the three N-glycosylation sites as occupied with Endo H-sensitive oligomannose N-glycans, consistent with its modification by TbSTT3B. Peptides encompassing the remaining two putative N-glycosylation sites, at Asn94 and Asn218, were not observed but their surrounding sequences would suggest that Asn94 is modified by TbSTT3A OST and likely to carry paucimannose structures and Asn218 is modified by TbSTT3B OST and likely to carry oligomannose structures (Table 1). Representations of the glycosylation of the ESAG6 and ESAG7 subunits of the TfR are shown in (Figure 4). The proteomics analysis of the TfR components (described above) also indicated that the principal ESAG6 and ESAG7 sequences present the purified TfR preparation corresponded to those deposited under accession numbers CAQ57442.1 and CAQ57441.1, respectively.

**Table 1:** Experimental and theoretical occupancy of TfR N-glycosylation sites.

Site^1^	Sequon	pI^2^	Endo H^3^	Predicted Major Glycan^4^
ESAG6,Asn010	ERNALNATAANKV	8.85	E+	Oligomannose, Man_5_GlcNAc_2_
ESAG6,Asn094	LEEMRNASALAAA	4.53	E−	Paucimannose, Man_4_GlcNAc_2_
ESAG6,Asn219	SPTRHNLTWGGGV	9.49	P+	Oligomannose, Man_5_GlcNAc_2_
ESAG6,Asn234	FGSYQNGSMYVEG	4.00	P−	Paucimannose, Man_4_GlcNAc_2_
ESAG6,Asn344	TILKSNYTAEPVR	8.26	E+	Oligomannose, Man_5_GlcNAc_2_
ESAG7,Asn010	ERNALNATAANKV	8.85	E+	Oligomannose, Man_5_GlcNAc_2_
ESAG7,Asn094	LEEMRNASALAAA	4.53	P−	Paucimannose, Man_4_GlcNAc_2_
ESAG7,Asn218	VCLNRNFTWGGGV	8.22	P+	Oligomannose, Man_5_GlcNAc_2_

1 Based on the predicted amino acid sequences for ESAG6 (GenBank: CAQ57442.1) and ESAG7 (GenBank: CAQ57441.1) minus their predicted 17-residue N-terminal signal peptides.

2 Predicted isoelectric point of the glycosylation sequon ±5 amino acid residues, as shown, calculated using ExPASy Compute pI/MW.

3 Experimentally determined (E) or theoretically predicted (P) sensitivity (+) or resistance (−) of the N-glycan to Endo H at the glycosylation site.

4 The major triantennary Endo H sensitive oligomannose Man_5_GlcNac_2_ structure is Manα1-3(Manα1-3(Manα1-6-Manα1-6)Manβ1-4GlcNAcβ1-4GlcNAc and the major biantennary Endo H resistant paucimannose Man_4_GlcNac_2_ structure is Manα1-2Manα1-3(Manα1-6Manβ1-4GlcNAcβ1-4GlcNAc.

### TfR binds indirectly glycoproteins that might, in turn, bind to tomato lectin

**Figure 5:**
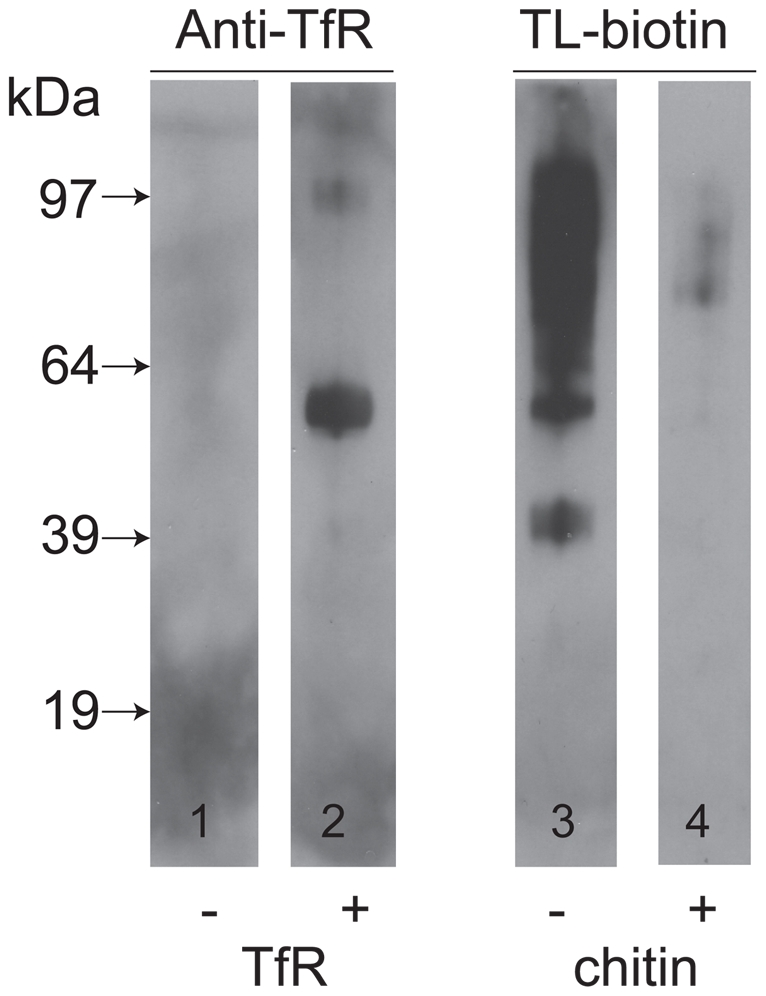
TfR does not bind directly to tomato lectin but binds to other glycoproteins. A ricin-binding glycoprotein fraction ([27] was purified from *T. brucei*, separated by SDS-PAGE and transferred to nitrocellulose. Identical lanes were incubated without (lane 1) or with (lane 2) purified TfR, followed by anti-TfR antibody, or with tomato lectin (TL) in the absence (lane 3) or presence (lane 4) of competing chitin hydrolysate. The positions of molecular weight markers are shown on the left.

Nolan and colleagues that have reported that TfR can be isolated from a trypanosome lysate with tomato lectin-Sepharose [38]. However, we did not identify any tomato lectin (TL) binding poly-LacNAc-containing N-glycans in either subunit of trypanosome TfR. We therefore entertained the possibility that TfR binds indirectly to TL through interaction with other glycoprotein(s) that do bear poly-LacNAc-containing N-glycans. To investigate this, we took osmotically lysed cells, depleted of VSG and TfR by the action of endogenous GPI-PLC on their GPI anchors, and isolated the total ricin-binding glycoprotein fraction, that includes the TL binding glycoproteins as a significant sub-set [27], and separated and immobilized them by SDS-PAGE and Western blot. The presence of TL-binding glycoproteins was confirmed by probing the blot with TL (Figure 5, lane 3) and the carbohydrate-specificity of this signal was confirmed by inhibition with chitin hydrolysate (Figure 5, lane 4). Identical blots were probed with anti-TfR antibodies before and after pre-incubation with purified TfR. Without pre-incubation with purified TfR, the anti-TfR blots were devoid of significant signal (Figure 5, lane 1), whereas with pre-incubation with purified TfR the anti-TfR blots showed two clear bands at apparent molecular weights of around 55 kDa and 97 kDa. From these data we conclude that TfR is able to bind to other glycoproteins that, in turn, can bind to ricin and therefore possibly also to TL.

### Molecular modeling of TfR in a VSG coat

**Figure 6:**
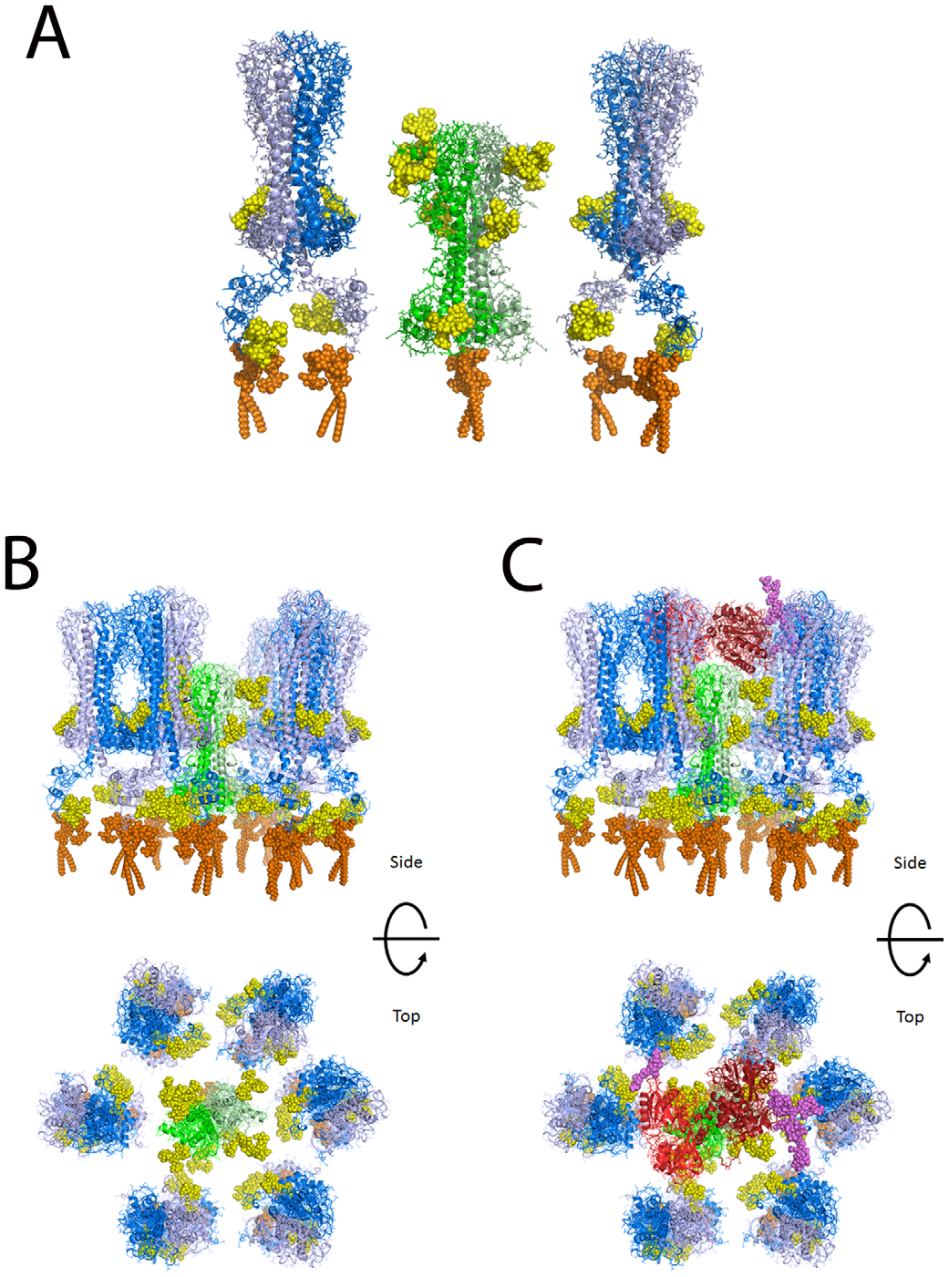
Molecular modeling of TfR in the VSG coat of *T. brucei*. (A) A single glycosylated ESAG6/ESAG7 TfR heterodimer is shown flanked by two glycosylated VSG homodimers. The molecular models suggest that TfR is likely to be recessed into the VSG surface coat. (B) Top and side views of a glycosylated TfR molecule surrounded by VSG molecules. (C) The same views as (B) but with a glycosylated transferrin molecule approaching the TfR. TfR: peptide chains – green; N-glycans – yellow; GPI anchor – orange. VSG: peptide chains – blue; N-glycans – yellow; GPI anchor – orange. Transferrin: peptide chains – red; N- and O-glycans – purple.

Based on the widely accepted assumption that *T. brucei* TfR has a similar tertiary structure and quaternary structure to the N-terminal domain of VSG [41], [42], for which there are crystallographic data [43], we have made a homology model of the ESAG6/ESAG7 heterodimer of TfR and added to this representative N-linked glycan structures, according to the data and predictions presented in this paper (Table 1 and Figure 4), and a GPI anchor [31]. VSG MITat1.2 was modeled based on the crystal structure of the N-terminal domain [43], the NMR structure of the C-terminal domain [44], and representative N-linked glycan and GPI anchor structures [2], [5], [35], [36]. The N-terminal and C-terminal domains were placed with relatively compact linkers between the two domains and between the C-terminal domain and the GPI anchor. With extended linkers the two domains could be displaced significantly further from the membrane. Human transferrin was modeled based on the structure of iron-bound human transferrin in complex with the human transferrin receptor [45] and representative N-linked [46] and O-linked [47] glycans. The comparison between the models of TfR and VSG MITat1.2 is shown (Figure 6A). A model of TfR surrounded by VSG molecules at their expected surface density [48] is also shown (Figure 6B). Into this model we have placed a model of glycosylated human transferrin, in the same orientation in which it docks to the human receptor [45] (Figure 6C). Although the TfR model is based on the specific ESAG6 and ESAG7 species found in our TfR preparation (accession numbers CAQ57442.1 and CAQ57441.1), the highly conserved amino acid sequences and glycosylation sites of the *T. brucei brucei* ESAG6 and ESAG7 families (Table S1 and Table S2) suggests that it would be reasonable to assume that this is a general model for all *T. brucei brucei* ESAG6/ESAG7 heterodimers.

## Discussion

As well as contributing to a three dimensional model of *T. brucei* TfR, the experimental data on N-glycosylation site occupancy for three of the five N-glycosylation sites of ESAG6 and one of the three for ESAG7 presented in this paper (Table 1; Figure 4) provide support for the model of a unique mechanism of protein N-glycosylation in *T. brucei* [34]–[37]. According to this model, *T. brucei* N-glycosylation sequons in relatively acidic environments (like Asn94 of ESAG6) co-translationally receive exclusively (Endo H-resistant) biantennary Man_5_GlcNAc_2_ through the action of an oligosaccharyltransferase (OST) encoded by the *TbSTT3A* gene whereas the remaining sites (like Asn10 and Asn344 of ESAG6 and Asn10 of ESAG7) are acted upon post-translationally by an OST encoded by the *TbSTT3B* gene and receive exclusively (Endo H-sensitive) triantennary oligomannose Man_9_GlcNAc_2_. Once transferred to protein, the biantennary Man_5_GlcNAc_2_ structure on the acidic sites may then be processed to paucimannose (Man_4_GlcNAc_2_ and Man_3_GlcNAc_2_) structures with the latter, in some cases, further elaborated to complex glycan structures. Apparently this further processing to complex glycans does not occur on ESAG6 or ESAG7, where Man_4_GlcNAc_2_ appears to be the predominant endo H-resistant structure. The triantennary oligomannose Man_9_GlcNAc_2_ structures at the non-acidic sites can only be maximally processed to the triantennary oligomannose Man_5_GlcNAc_2_ structure, which appears to be the predominant endo H-sensitive structure on ESAG6 and ESAG7.

Another recent analysis of the single N-glycan of VSG MITat.1.8 also supported the model in [34]. In this case, a single acidic N-glycosylation site at Asn59 was found to be occupied exclusively by a biantennery complex N-glycan structure of Galβ1-4GlcNAcβ1-2Manα1-3(Galβ1-4GlcNAcβ1-2Manα1-6)Manβ1-4GlcNAcβ1-4GlcNAc. Presumably, and in contrast to the VSG MITat.1.8 example, the acidic TfR N-glycosylation sites fail to be processed beyond the trimming of up to two α1-2-linked mannose residues due to steric constraints, reducing access by α-mannosidases and preventing subsequent access by βGlcNAc-transferases.

It was suggested by Nolan and colleagues that *T. brucei* TfR contains poly-LacNAc glycans because ESAG6 and ESAG7 in whole cell detergent lysates bound to tomato lectin (TL) beads [38]. These authors further suggested a tentative model for endocytosis in trypanosomes, postulating an interaction between poly-LacNAc N-glycans on TfR (and other receptors) and a protein in the flagellar pocket with an extracellular TL lectin-like domain and a cytoplasmic domain that interacts with the machinery of the endocytic pathway. This model was supported by an approximately 5-fold reduction in transferrin endocytic rate when trypanosomes were incubated in 15 mM each of tri-*N*-acetyl-chitotriose and tetra-*N*-acetyl-chitotetraose. However, our data show that TfR does not contain any poly-LacNAc structures. Therefore, a direct link between receptor-linked poly-LacNAc glycans and endocytic machinery can be ruled out. However, we have shown here that TfR is able to bind to immobilized ricin-binding glycoproteins. Since the TL-binding glycoproteins of *T. brucei* are a sub-set of the ricin-binding fraction [27], these data may explain why TfR was found in the TL-binding fraction [38], *i.e.*, through the non-covalent association of TfR with other glycoproteins. It should be pointed out that the association seen in the TfR overlay experiment could be through protein-protein and/or protein-carbohydrate interaction(s) and that, relevant to possible protein-carbohydrate interactions, the glycoproteins in the ricin-binding fraction contain oligomannose and paucimannose glycans as well as conventional complex and poly-LacNAc containing N-glycans. The latter two classes of glycan bind directly to ricin while the former are present because many glycoproteins contain a mixture of both oligomannose and/or paucimannose and complex and/or poly-LacNAc glycans attached to different glycosylation sites in the same polypeptide. While the indirect association of TfR with other glycoproteins could still be relevant for poly-LacNAc-mediated endocytosis in theory, the normal *in vitro* growth rate of bloodstream form trypanosomes under TbSTT3A RNAi knockdown [34], when the synthesis of almost all complex N-glycans (including poly-LacNAc glycans) is abrogated, also brings the model of poly-LacNAc-mediated endocytosis into question. We therefore suggest that we should return to a null hypothesis: That transferrin, captured by the parasite TfR embedded in the VSG coat, is endocytosed constitutively in clathrin-coated vesicles and that the extremely rapid turnover of the flagellar pocket membrane in bloodstream form *T. brucei* [49], [50] provides a sufficient rate of uptake of this (and other) essential macromolecular nutrients from host serum.

The molecular modeling of TfR alongside VSG shows that TfR is predicted to sit low in the VSG coat. However, the N-glycans of TfR significantly increase the surface area occupied by TfR compared with VSG. This may be physiologically relevant since the TfR glycans may contribute to protecting the underlying plasma membrane from lytic host serum components while providing sufficient space to allow access of the 80 kDa bi-lobed transferrin glycoprotein, regardless of the relative orientations of the receptor and the ligand (which are currently unknown) when binding takes place. Thus, the widest diameter of transferrin is significantly larger than that of aglycosyl TfR but similar to that of glycosylated TfR. Previously, we had speculated that since the single dimyristoyl-GPI anchor of the ESAG6/ESAG7 TfR heterodimer (as compared to the twin dimyristoyl-GPI anchors of VSG homodimers) would lead to relatively weak association of TfR with the flagellar pocket membrane [51], this might allow TfR to leave the membrane and dock with transferrin in the fluid phase of the flagellar pocket [31]. While the molecular modeling presented here does not altogether rule that model out, it does suggest that the TfR does not *de facto* have to leave the membrane to dock with its ligand.

Finally, one would predict that transferrin/TfR accessibility at the flagellar pocket membrane is under extreme spatial constraint to prevent complement activation by the underlying plasma membrane. In other words, one would predict that TfR should be able make sufficient space within the VSG coat to allow transferrin to approach and be captured but without exposing significantly more underlying plasma membrane than found throughout the rest of the VSG coat. This tuning of the space occupied by TfR appears to be satisfied by N-glycosylation of both of its subunits and it may explain why TfR has so many N-glycosylation sites (eight) compared to the structurally-related VSGs, which generally have only two or four N-glycans per VSG dimer [4].

## Materials and Methods

### Ethics statement

Rodents were used to propagate sufficient *T. brucei* parasites for the purification of sufficient transferrin receptor for high-sensitivity structural analyses. The animal procedures were carried out according the United Kingdom Animals (Scientific Procedures) Act 1986 and according to specific protocols approved by The University of Dundee Ethics Committee and as defined and approved in the UK Home Office Project License PPL 60/3836 held by MAJF.

### Purification of TfR

The transferrin receptor was purified from blood stream form trypanosomes as previously described by Mehlert and Ferguson [31] using affinity chromatography with transferrin-Sepharose which was first described in [39].

### Exoglycosidase digestion, SDS-PAGE and western blotting of TfR

Exoglycosidase digests were carried out using both N-glycanase F and Endoglycosidase H as described in Izquierdo et al [34]. The exoglycosidase digests were analyzed by reducing SDS-PAGE with 4–12% gradient gels (Invitrogen), using MOPs buffer and then Western blotting onto nitrocellulose (GE Healthcare) as in [28]. After blocking and incubating in rabbit polyclonal anti-transferrin receptor (kindly supplied by Dietmer Steverding) at the dilution of 1 in 1000 then washing several times in blocking buffer, the membranes were incubated in Anti-rabbit HRP at a dilution of 1 in 20,000. After further washing visualization of the bands was achieved using ECL reagents (GE Healthcare).

### Lectin blotting of TfR

SDS PAGE and Western blotting was carried out as above and then the membranes were stained using lectins as described in [34]. All lectin-biotin conjugates were obtained from Vector laboratories. Concanavalin A conjugated to biotin was used at a dilution of 1 in 3,000 (with or without 0.5 M α-methyl-mannose). Ricin-biotin was used at a dilution of 1 in 3,000 (with or without 10 mg/ml galactose and 10 mg/ml lactose), tomato lectin-biotin conjugate diluted was used at a dilution of 1 in 10,000 (with or without chitin hydrolysate, Vector Laboratories, at a dilution of 1 in 10), ErCr lectin was used at a dilution of 1 in 3,000 (with or without 200 mM lactose). The blots were washed extensively after being incubated with the lectin solutions and were incubated in streptavidin-HRP obtained from Sigma Aldrich and diluted to 1 in 10,000. Bands were visualized using ECL reagents as above.

### Release, radiolabeling and analysis of TfR N-glycans

The N-glycans of the trypanosomal heterodimeric transferrin receptor were released by PNGase-F and labeled with sodium borotritiide following the method described in [28]. After extensive cleanup steps to remove any contaminating tritiated material [28] the ^3^H-labeled glycans were analyzed by HPTLC [Merck silica gel 60] and fractionated by HPAEC as described in [28] and fractions were pooled according to the amount of radioactivity after 10% was used for scintillation counting. Some of the pools were digested using the broad specificity alpha mannosidase extracted from *Canavalia ensiformis* (jack beans) (Sigma-Aldrich) and the α1-2 specific alpha mannosidase extracted from *Aspergillus saitoi* (Prozyme), as described in [2]. After digestion the samples were desalted using a mixed bed column as described in [2] and then analyzed again by HTPLC as above. The HTPLC plates were run 3 times in butanol ∶ methanol ∶ water, 4 ∶ 4 ∶ 3 (v/v), with drying between each run, then dried, sprayed with En^3^Hance (Perkin Elmer) and fluorographed with intensifying screens for up to 8 weeks at −80°C.

### Analysis of TfR N-glycosylation site occupancy by LC-MS/MS

Samples of transferrin receptor were digested with Endoglycosidase H followed by PNGaseF digestion (Roche), then analyzed by SDS-PAGE as above. Following staining with Simply Blue (Sigma-Aldrich) the bands corresponding to ESAG6 and 7 were cut out and subjected to proteomic analysis. An aliquot of the tryptic digest was analyzed by LC-MS on an LTQ Orbitrap XL (Thermo) using a Dionex 3000 Nano-LC as in [34]. The resulting data were analyzed using Mascot and the *T. brucei* geneDB protein database using variable modifications of N-acetylated glucosamine modification of Asn, which would signify an Endoglycosidase H sensitive site, and deamidation of Asn to Asp, which would signify an Endoglycosidase H resistant site, as described in [34].

### TfR overlay experiments

A ricin-binding total glycoprotein fraction from bloodstream form *T. brucei* [27] was subjected to SDS-PAGE and transfer to nitrocellulose. These blots were probed with tomato lectin (with and without chitin hydrolysate inhibitor), as described above, or with or without purified TfR (approximately 0.2 µg/ml in phosphate buffered saline). The latter blots were subsequently probed with anti-TfR antibody with ECL detection, as described above.

### Molecular modeling

Molecular modeling was performed on a Silicon Graphics Fuel workstation using InsightII and Discover software (Accelrys Inc.,San Diego, USA). Figures were produced using PyMol (The PyMOL Molecular Graphics System, Schrödinger, LLC). Protein structures used for modeling were obtained from the pdb database [52].

The homology model of *T. brucei* TfR was based on crystal structure of VSG MITat1.2 (pdb code - 1vsg [43]). The sequence alignment between ESAG6, ESAG7 and VSG was based on [44], modified to take account of the protein tertiary structure and the additional disulphide bonds present. The formation of the disulphide bonds in ESAG6 and ESAG7 between residues equivalent to residues 62 and 286 in MITat1.2 required a distortion of the helix starting at residue 61 and a rearrangement of the loop-containing residue 286. The additional disulphide bond in ESAG7 between residues equivalent to residues 203 and 220 in MITat1.2 could be accommodated with no alteration in the secondary or tertiary protein structure. The model of VSG MITat1.2 was based on the crystal structure of the N-terminal domain (pdb code – 1vsg [43]) and the NMR structure of the C-terminal domain (pdb code – 1xu6 [44]). The C-terminal domain was placed directly below the N-terminal domain [44] to allow for the dense packing of the N-terminal domains on the trypanosome surface [48]. The linkers between the two domains and between the C-terminal domain and the GPI anchor were modeled as relatively compact random loops. The model of human transferrin was based on the structure of iron-bound transferrin in complex with the human transferrin receptor (pdb code – 1suv [45]), N-linked and O-linked glycan structures and GPI anchors were added to all models as appropriate. The structure of the glycans were generated using the database of glycosidic linkage conformations [52] and *in vacuo* energy minimisation to relieve unfavorable steric interactions. The Asn-GlcNAc linkage conformations were based on the observed range of crystallographic values [53], [54] the torsion angles around the Asn Cα-Cβ and Cβ-Cγ bonds then being adjusted to eliminate unfavorable steric interactions between the glycans and the protein surface.

### Accession numbers used in this study

The following GenBank protein sequence accession numbers were used in this study: CAQ57442.1 and CAQ57441.1. The following Protein Data Bank (pdb) files were used in this study: 1vsg, 1xu6, 1suv.

## Supporting Information

Figure S1 Purified TfR does not react with tomato lectin (TL). Aliquots of purified *T. brucei* TfR (lanes 1, 2, and 3) and of the *T. brucei* ricin-binding glycoprotein fraction (a positive control for TL blotting, lanes 4 and 5) were separated by SDS-PAGE, transferred to nitrocellulose and subjected to blotting with anti-TfR antibody (lane 1) or TL (lanes 2–5) in the absence (−) or presence (+) of the TL inhibitor chitin hydrolysate. The positions of molecular weight markers are indicated for each group of blots.
(DOC)

Figure S2 HPTLC analysis of N-glycans released from TfR and labeled by NaB[^3^H]_4_-reduction alongside known glycan standards and structures released from other characterized glycoproteins. All glycoprotein samples were treated with PNGaseF at the same time and with the same reagents. Similarly, all glycan samples (derived from glycoproteins and pure glycans supplied by Dextra Labs) were reduced at the same time with the same reagents. Aliquots of all radiolabeled total glycan fractions were separated on the same HPTLC plate and detected by fluorography, as described in Materials and Methods. The radiolabeled glycans were derived from: bovine ribonuclease B (RNaseB) (lane 1), pure Man_6_GlcNAc_2_ (M6) (lane 2), pure Man_8_GlcNAc_2_ (M8) (lane 3), *T. brucei* VSG MITat1.4 (lane 4), *T. brucei* TfR (lane5), *T.brucei* VSG MITat1.7 (lane 6) and pure (Gal-GlcNAc)_2_Man_3_GlcNAc_2_ (NA2) (lane 7). The sample in lane 5 is that shown in Figure 3A. Bovine pancreatic ribonuclease B contains exclusively oligomannose structures ranging from Man_9_GlcNAc_2_ to Man_5_GlcNAc_2_ (M9-M5) [55] as does VSG MITat1.4 [6]. The assignments for the VSG MITat1.7 glycans are based on the structures published by Zamze *et al.* in [5].
(DOC)

Figure S3 Dionex HPAEC separation of radiolabeled N-glycans. PNGase F-released and NaB^3^H_4_-reduced N-glycans were separated [28] by Dionex high-pH anion exchange chromatography on a Dionex CarboPac PA-100 column (2 mm by 250 mm). The column was equilibrated with 98% buffer A (100 mM NaOH) and 2% buffer B (380 mM sodium acetate in 100 mM NaOH) for 20 min at a flow rate of 0.25 ml/min. N-glycans were separated using a linear gradient of 2 to 25% buffer B over 40 min at 0.6 ml/min. The collection of 0.25 ml fractions was started after 3 min and aliquots (10%) of each were taken for liquid scintillation counting. The fractions corresponding to *peaks a, b and c* are indicated.
(DOC)

Table S1 CLUSTAL 2.1 multiple sequence alignment of 21 *T. brucei brucei* ESAG6 sequences. The sequences in general and the N-glycosylation sites (in bold) in particular are highly conserved in the different ESAG6 family members.
(DOC)

Table S2 CLUSTAL 2.1 multiple sequence alignment of 14 *T. brucei brucei* ESAG7 sequences. The sequences in general and the N-glycosylation sites (in bold) in particular are highly conserved in the different ESAG6 family members.
(DOC)

## References

[1] Ferguson MA, Homans SW, Dwek RA, Rademacher TW (1988). Glycosyl-phosphatidylinositol moiety that anchors *Trypanosoma brucei* variant surface glycoprotein to the membrane.. Science.

[2] Mehlert A, Richardson JM, Ferguson MA (1998). Structure of the glycosylphosphatidylinositol membrane anchor glycan of a class-2 variant surface glycoprotein from *Trypanosoma brucei*.. J Mol Biol.

[3] Mehlert A, Sullivan L, Ferguson MA (2010). Glycotyping of *Trypanosoma brucei* variant surface glycoprotein MITat1.8.. Mol Biochem Parasitol.

[4] Mehlert A, Zitzmann N, Richardson JM, Treumann A, Ferguson MA (1998). The glycosylation of the variant surface glycoproteins and procyclic acidic repetitive proteins of *Trypanosoma brucei*.. Mol Biochem Parasitol.

[5] Zamze S, Ashford DA, Wooten EW, Rademacher TW, Dwek RA (1991). Structural characterization of the asparagine-linked oligosaccharides from *Trypanosoma brucei* type II and type III variant surface glycoproteins.. J Biol Chem.

[6] Zamze SE, Wooten EW, Ashford DA, Ferguson MA, Dwek RA (1990). Characterisation of the asparagine-linked oligosaccharides from *Trypanosoma brucei* type-I variant surface glycoproteins.. Eur J Biochem.

[7] Stockdale C, Swiderski MR, Barry JD, McCulloch R (2008). Antigenic variation in *Trypanosoma brucei*: joining the DOTs.. PLoS Biol.

[8] Ziegelbauer K, Overath P (1993). Organization of two invariant surface glycoproteins in the surface coat of *Trypanosoma brucei*.. Infect Immun.

[9] Ziegelbauer K, Overath P (1992). Identification of invariant surface glycoproteins in the bloodstream stage of *Trypanosoma brucei*.. J Biol Chem.

[10] Nozaki T, Haynes PA, Cross GA (1996). Characterization of the *Trypanosoma brucei* homologue of a *Trypanosoma cruzi* flagellum-adhesion glycoprotein.. Mol Biochem Parasitol.

[11] Steverding D, Stierhof YD, Chaudhri M, Ligtenberg M, Schell D (1994). ESAG 6 and 7 products of *Trypanosoma brucei* form a transferrin binding protein complex.. Eur J Cell Biol.

[12] Kelley RJ, Alexander DL, Cowan C, Balber AE, Bangs JD (1999). Molecular cloning of p67, a lysosomal membrane glycoprotein from *Trypanosoma brucei*.. Mol Biochem Parasitol.

[13] Lingnau A, Zufferey R, Lingnau M, Russell DG (1999). Characterization of tGLP-1, a Golgi and lysosome-associated, transmembrane glycoprotein of African trypanosomes.. J Cell Sci.

[14] Guther ML, Prescott AR, Ferguson MA (2003). Deletion of the GPIdeAc gene alters the location and fate of glycosylphosphatidylinositol precursors in *Trypanosoma brucei*.. Biochemistry.

[15] Engstler M, Weise F, Bopp K, Grunfelder CG, Gunzel M (2005). The membrane-bound histidine acid phosphatase TbMBAP1 is essential for endocytosis and membrane recycling in *Trypanosoma brucei*.. J Cell Sci.

[16] Roditi I, Furger A, Ruepp S, Schurch N, Butikofer P (1998). Unravelling the procyclin coat of *Trypanosoma brucei*.. Mol Biochem Parasitol.

[17] Acosta-Serrano A, Cole RN, Mehlert A, Lee MG, Ferguson MA (1999). The procyclin repertoire of *Trypanosoma brucei*. Identification and structural characterization of the Glu-Pro-rich polypeptides.. J Biol Chem.

[18] Roper JR, Guther ML, Macrae JI, Prescott AR, Hallyburton I (2005). The suppression of galactose metabolism in procylic form *Trypanosoma brucei* causes cessation of cell growth and alters procyclin glycoprotein structure and copy number.. J Biol Chem.

[19] Vassella E, Butikofer P, Engstler M, Jelk J, Roditi I (2003). Procyclin null mutants of *Trypanosoma brucei* express free glycosylphosphatidylinositols on their surface.. Mol Biol Cell.

[20] Guther ML, Beattie K, Lamont DJ, James J, Prescott AR (2009). Fate of glycosylphosphatidylinositol (GPI)-less procyclin and characterization of sialylated non-GPI-anchored surface coat molecules of procyclic-form *Trypanosoma brucei*.. Eukaryot Cell.

[21] Guther ML, Lee S, Tetley L, Acosta-Serrano A, Ferguson MA (2006). GPI-anchored proteins and free GPI glycolipids of procyclic form *Trypanosoma brucei* are nonessential for growth, are required for colonization of the tsetse fly, and are not the only components of the surface coat.. Mol Biol Cell.

[22] Hertz-Fowler C, Figueiredo LM, Quail MA, Becker M, Jackson A (2008). Telomeric expression sites are highly conserved in *Trypanosoma brucei*.. PloS One.

[23] Ansorge I, Steverding D, Melville S, Hartmann C, Clayton C (1999). Transcription of ‘inactive’ expression sites in African trypanosomes leads to expression of multiple transferrin receptor RNAs in bloodstream forms.. Mol Biochem Parasitol.

[24] Steverding D (2003). The significance of transferrin receptor variation in *Trypanosoma brucei*.. Trends Parasitol.

[25] van Luenen HG, Kieft R, Mussmann R, Engstler M, ter Riet B (2005). Trypanosomes change their transferrin receptor expression to allow effective uptake of host transferrin.. Mol Microbiol.

[26] Treumann A, Zitzmann N, Hulsmeier A, Prescott AR, Almond A (1997). Structural characterisation of two forms of procyclic acidic repetitive protein expressed by procyclic forms of *Trypanosoma brucei*.. J Mol Biol.

[27] Atrih A, Richardson JM, Prescott AR, Ferguson MA (2005). *Trypanosoma brucei* glycoproteins contain novel giant poly-N-acetyllactosamine carbohydrate chains.. J Biol Chem.

[28] Izquierdo L, Atrih A, Rodrigues JA, Jones DC, Ferguson MA (2009). *Trypanosoma brucei* UDP-glucose:glycoprotein glucosyltransferase has unusual substrate specificity and protects the parasite from stress.. Eukaryot Cell.

[29] Hwa KY, Khoo KH (2000). Structural analysis of the asparagine-linked glycans from the procyclic *Trypanosoma brucei* and its glycosylation mutants resistant to Concanavalin A killing.. Mol Biochem Parasitol.

[30] Acosta-Serrano A, O'Rear J, Quellhorst G, Lee SH, Hwa KY (2004). Defects in the N-linked oligosaccharide biosynthetic pathway in a *Trypanosoma brucei* glycosylation mutant.. Eukaryot Cell.

[31] Mehlert A, Ferguson MA (2007). Structure of the glycosylphosphatidylinositol anchor of the *Trypanosoma brucei* transferrin receptor.. Mol Biochem Parasitol.

[32] Steverding D, Stierhof YD, Fuchs H, Tauber R, Overath P (1995). Transferrin-binding protein complex is the receptor for transferrin uptake in *Trypanosoma brucei*.. J Cell Biol.

[33] Steverding D (1998). Bloodstream forms of *Trypanosoma brucei* require only small amounts of iron for growth.. Parasitol Res.

[34] Izquierdo L, Schulz BL, Rodrigues JA, Guther ML, Procter JB (2009). Distinct donor and acceptor specificities of *Trypanosoma brucei* oligosaccharyltransferases.. EMBO J.

[35] Manthri S, Guther ML, Izquierdo L, Acosta-Serrano A, Ferguson MA (2008). Deletion of the TbALG3 gene demonstrates site-specific N-glycosylation and N-glycan processing in *Trypanosoma brucei*.. Glycobiology.

[36] Jones DC, Mehlert A, Guther ML, Ferguson MA (2005). Deletion of the glucosidase II gene in *Trypanosoma brucei* reveals novel N-glycosylation mechanisms in the biosynthesis of variant surface glycoprotein.. J Biol Chem.

[37] Bangs JD, Doering TL, Englund PT, Hart GW (1988). Biosynthesis of a variant surface glycoprotein of *Trypanosoma brucei*. Processing of the glycolipid membrane anchor and N-linked oligosaccharides.. J Biol Chem.

[38] Nolan DP, Geuskens M, Pays E (1999). N-linked glycans containing linear poly-N-acetyllactosamine as sorting signals in endocytosis in *Trypanosoma brucei*.. Curr Biol.

[39] Steverding D, Overath P (1996). *Trypanosoma brucei* with an active metacyclic variant surface gene expression site expresses a transferrin receptor derived from esag6 and esag7.. Mol Biochem Parasitol.

[40] Itakura Y, Nakamura-Tsuruta S, Kominami J, Sharon N, Kasai K (2007). Systematic comparison of oligosaccharide specificity of *Ricinus communis* agglutinin I and Erythrina lectins: a search by frontal affinity chromatography.. J Biochem.

[41] Salmon D, Hanocq-Quertier J, Paturiaux-Hanocq F, Pays A, Tebabi P (1997). Characterization of the ligand-binding site of the transferrin receptor in *Trypanosoma brucei* demonstrates a structural relationship with the N-terminal domain of the variant surface glycoprotein.. EMBO J.

[42] Carrington M, Boothroyd J (1996). Implications of conserved structural motifs in disparate trypanosome surface proteins.. Mol Biochem Parasitol.

[43] Freymann D, Down J, Carrington M, Roditi I, Turner M (1990). 2.9 A resolution structure of the N-terminal domain of a variant surface glycoprotein from *Trypanosoma brucei*.. J Mol Biol.

[44] Chattopadhyay A, Jones NG, Nietlispach D, Nielsen PR, Voorheis HP (2005). Structure of the C-terminal domain from *Trypanosoma brucei* variant surface glycoprotein MITat1.2.. J Biol Chem.

[45] Cheng Y, Zak O, Aisen P, Harrison SC, Walz T (2004). Structure of the human transferrin receptor-transferrin complex.. Cell.

[46] Fu D, van Halbeek H (1992). N-glycosylation site mapping of human serotransferrin by serial lectin affinity chromatography, fast atom bombardment-mass spectrometry, and 1H nuclear magnetic resonance spectroscopy.. Anal Biochem.

[47] van Rooijen JJ, Jeschke U, Kamerling JP, Vliegenthart JF (1998). Expression of N-linked sialyl Le(x) determinants and O-glycans in the carbohydrate moiety of human amniotic fluid transferrin during pregnancy.. Glycobiology.

[48] Mehlert A, Bond CS, Ferguson MA (2002). The glycoforms of a *Trypanosoma brucei* variant surface glycoprotein and molecular modeling of a glycosylated surface coat.. Glycobiology.

[49] Engstler M, Thilo L, Weise F, Grunfelder CG, Schwarz H (2004). Kinetics of endocytosis and recycling of the GPI-anchored variant surface glycoprotein in *Trypanosoma brucei*.. J Cell Sci.

[50] Overath P, Engstler M (2004). Endocytosis, membrane recycling and sorting of GPI-anchored proteins: *Trypanosoma brucei* as a model system.. Mol Microbiol.

[51] Schwartz KJ, Peck RF, Tazeh NN, Bangs JD (2005). GPI valence and the fate of secretory membrane proteins in African trypanosomes.. J Cell Sci.

[52] Berman HM, Westbrook J, Feng Z, Gilliland G, Bhat TN (2000). The Protein Data Bank.. Nucleic Acids Res.

[53] Wormald MR, Petrescu AJ, Pao YL, Glithero A, Elliott T (2002). Conformational studies of oligosaccharides and glycopeptides: complementarity of NMR, X-ray crystallography, and molecular modelling.. Chem Rev.

[54] Petrescu AJ, Milac AL, Petrescu SM, Dwek RA, Wormald MR (2004). Statistical analysis of the protein environment of N-glycosylation sites: implications for occupancy, structure, and folding.. Glycobiology.

[55] Rudd PM, Joao HC, Coghill E, Fiten P, Saunders MR (1994). Glycoforms modify the dynamic stability and functional activity of an enzyme.. Biochemistry.

