# Supplementary material for: Modeling of the N-Glycosylated Transferrin Receptor Suggests How Transferrin Binding Can Occur within the Surface Coat of Trypanosoma brucei
Source: PLoS Pathog. 2012 Apr 5;8(4):e1002618. doi: 10.1371/journal.ppat.1002618 (PMC3320590; doi:10.1371/journal.ppat.1002618)
Supplement: Figure S1 — Purified TfR does not react with tomato lectin (TL). Aliquots of purified T. brucei TfR (lanes 1, 2, and 3) and of the T. brucei ricin-binding glycoprotein fraction (a positive control for TL blotting, lanes 4 and 5) were separated by SDS-PAGE, transferred to nitrocellulose and subjected to blotting with anti-TfR antibody (lane 1) or TL (lanes 2–5) in the absence (−) or presence (+) of the TL inhibitor chitin hydrolysate. The positions of molecular weight markers are indicated for each group of blots. (DOC) [file ppat.1002618.s001.doc]

**Figure S1.** Purified TfR does not react with tomato lectin (TL).

Aliquots of purified *T. brucei* TfR (lanes 1, 2, and 3) and of the *T. brucei* ricin-binding glycoprotein fraction (, a positive control for TL blotting (lanes 4 and 5) were separated by SDS-PAGE, transferred to nitrocellulose and subjected to blotting with anti-TfR antibody (lane 1) or TL (lanes 2-5) in the absence (-) or presence (+) of the TL inhibitor chitin hydrolysate. The positions of molecular weight markers are indicated for each group of blots.
