# Supplementary material for: Modeling of the N-Glycosylated Transferrin Receptor Suggests How Transferrin Binding Can Occur within the Surface Coat of Trypanosoma brucei
Source: PLoS Pathog. 2012 Apr 5;8(4):e1002618. doi: 10.1371/journal.ppat.1002618 (PMC3320590; doi:10.1371/journal.ppat.1002618)
Supplement: Figure S2 — HPTLC analysis of N-glycans released from TfR and labeled by NaB[3H]4-reduction alongside known glycan standards and structures released from other characterized glycoproteins. All glycoprotein samples were treated with PNGaseF at the same time and with the same reagents. Similarly, all glycan samples (derived from glycoproteins and pure glycans supplied by Dextra Labs) were reduced at the same time with the same reagents. Aliquots of all radiolabeled total glycan fractions were separated on the same HPTLC plate and detected by fluorography, as described in Materials and Methods. The radiolabeled glycans were derived from: bovine ribonuclease B (RNaseB) (lane 1), pure Man6GlcNAc2 (M6) (lane 2), pure Man8GlcNAc2 (M8) (lane 3), T. brucei VSG MITat1.4 (lane 4), T. brucei TfR (lane5), T.brucei VSG MITat1.7 (lane 6) and pure (Gal-GlcNAc)2Man3GlcNAc2 (NA2) (lane 7). The sample in lane 5 is that shown in Figure 3A. Bovine pancreatic ribonuclease B contains exclusively oligomannose structures ranging from Man9GlcNAc2 to Man5GlcNAc2 (M9-M5) [55] as does VSG MITat1.4 [6]. The assignments for the VSG MITat1.7 glycans are based on the structures published by Zamze et al. in [5]. (DOC) [file ppat.1002618.s002.doc]

**Figure S2.** HPTLC analysis of N-glycans released from TfR and labeled by NaB[^3^H]_4_-reduction alongside known glycan standards and structures released from other characterized glycoproteins.

All glycoprotein samples were treated with PNGaseF at the same time and with the same reagents. Similarly, all glycan samples (derived from glycoproteins and pure glycans supplied by Dextra Labs) were reduced at the same time with the same reagents. Aliquots of all radiolabelled total glycan fractions were separated on the same HPTLC plate and detected by fluorography, as described in Materials and Methods. The radiolabelled glycans were derived from: bovine ribonuclease B (RNaseB) (lane 1), pure Man_6_GlcNAc_2_ (M6) (lane 2), pure Man_8_GlcNAc_2_  (M8) (lane 3), *T.brucei* VSG MITat1.4 (lane 4), *T.brucei* TfR (lane5), *T.brucei* VSG MITat1.7 (lane 6) and pure (Gal-GlcNAc)_2_Man_3_GlcNAc_2_ (NA2) (lane 7). The sample in lane 5 is that shown in Figure 3A. Bovine pancreatic ribonuclease B contains exclusively oligomannose structures ranging from Man_9_GlcNAc_2_ to Man_5_GlcNAc_2_ (M9-M5) (Rudd *et al* (1994) *Biochemistry* *33*, 17-22) as does VSG MITat1.4 [6]. The assignments for the VSG MITat1.7 glycans are based on the structures published by Zamze *et al*. in [5].
