# Supplementary material for: Modeling of the N-Glycosylated Transferrin Receptor Suggests How Transferrin Binding Can Occur within the Surface Coat of Trypanosoma brucei
Source: PLoS Pathog. 2012 Apr 5;8(4):e1002618. doi: 10.1371/journal.ppat.1002618 (PMC3320590; doi:10.1371/journal.ppat.1002618)
Supplement: Figure S3 — Dionex HPAEC separation of radiolabeled N-glycans. PNGase F-released and NaB3H4-reduced N-glycans were separated [28] by Dionex high-pH anion exchange chromatography on a Dionex CarboPac PA-100 column (2 mm by 250 mm). The column was equilibrated with 98% buffer A (100 mM NaOH) and 2% buffer B (380 mM sodium acetate in 100 mM NaOH) for 20 min at a flow rate of 0.25 ml/min. N-glycans were separated using a linear gradient of 2 to 25% buffer B over 40 min at 0.6 ml/min. The collection of 0.25 ml fractions was started after 3 min and aliquots (10%) of each were taken for liquid scintillation counting. The fractions corresponding to peaks a, b and c are indicated. (DOC) [file ppat.1002618.s003.doc]

**Figure S3.** Dionex HPAEC separation of radiolabelled N-glycans.


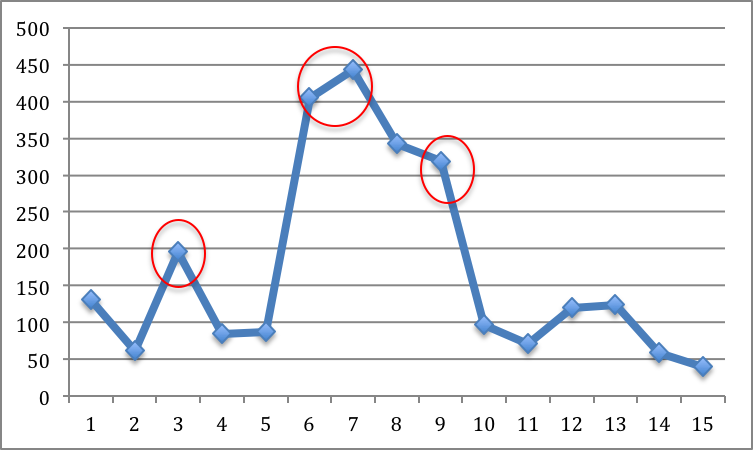


Peak a

Peak b

Peak c

Radio-

activity

(cpm)

Fraction number

PNGase F-released and NaB^3^H_4_-reduced N-glycans were separated (28) by Dionex high-pH anion exchange chromatography on a Dionex CarboPac PA-100 column (2 mm by 250 mm). The column was equilibrated with 98% buffer A (100 mM NaOH) and 2% buffer B (380 mM sodium acetate in 100 mM NaOH) for 20 min at a flow rate of 0.25

ml/min. N-glycans were separated using a linear gradient of 2 to 25% buffer B

over 40 min at 0.6 ml/min. The collection of 0.25 ml fractions was started after 3 min and aliquots (10%) of each were taken for liquid scintillation counting. The fractions corresponding to *peaks a, b and c* are indicated.
