# Supplementary material for: Modeling of the N-Glycosylated Transferrin Receptor Suggests How Transferrin Binding Can Occur within the Surface Coat of Trypanosoma brucei
Source: PLoS Pathog. 2012 Apr 5;8(4):e1002618. doi: 10.1371/journal.ppat.1002618 (PMC3320590; doi:10.1371/journal.ppat.1002618)
Supplement: Table S1 — CLUSTAL 2.1 multiple sequence alignment of 21 T. brucei brucei ESAG6 sequences. The sequences in general and the N-glycosylation sites (in bold) in particular are highly conserved in the different ESAG6 family members. (DOC) [file ppat.1002618.s004.doc]

**Table S1. CLUSTAL 2.1 multiple sequence alignment of 21 T. brucei brucei ESAG6 sequences.**

The sequences in general and the N-glycosylation sites (in bold) in particular are highly conserved in the different ESAG6 family members.

gi|197091027|gb|ACH41867.1| -MRFWFVLLAVLGKETYA-YYENERNAL**NAT**AANKVCALSTYLKGIAHRV 48

gi|197091011|gb|ACH41859.1| -MRFLFVLLAVLGKETYA-YYENERNAL**NAT**AANKVCALSTYLKGIAHRV 48

gi|197090997|gb|ACH41852.1| MMRFLFVLLALLRKETHANYYENERNAL**NAT**AANKVCGLSTYLKGIAHRV 50

gi|197090935|gb|ACH41831.1| -MRFLFVLLALLGKETHANYYENERNAL**NAT**AANKVCGLSTYLKGIAHRV 49

gi|197091003|gb|ACH41855.1| -MRFLFVLLALLGKKTHAYY-ENERNAL**NAT**AANKVCGLSTYLKGIAHRV 48

gi|197091001|gb|ACH41854.1| -MRFLFVLLALLGKKTHAYY-ENERNAL**NAT**AANKVCGLSTYLKGIAHRV 48

gi|197091015|gb|ACH41861.1| -MRFLFVLLALLGKKTHAYY-ENERNAL**NAT**AANKVCGLSTYLKGIAHRV 48

gi|189094728|emb|CAQ57403.1| -MRFLFVLLALLGKEIYAY--ENERNAL**NAT**AANKVCGLSTYLKGIAHRV 47

gi|197090991|gb|ACH41849.1| -MRFWFVLLALLGKETYAYY-ENERNAL**NAT**AANKVCGLSTYLKGIAHRV 48

gi|197091023|gb|ACH41865.1| -MRFWFVLLALLGKEIYAY--ENERNAL**NAT**AANKVCGLSTYLKGIAHRV 47

gi|197091005|gb|ACH41856.1| -MRFLFVLLALLGKETYAYY-ENERNAL**NAT**AANKVCGLSTYLKGIAHRV 48

gi|189094648|emb|CAQ57310.1| -MRFWFVLLALLGKEIYAY--ENERNAL**NAT**AANKVCGLSTYLKGIAHRV 47

gi|197091025|gb|ACH41866.1| -MRFLFLLLVLLGKKTHANYYENKRNAL**NAT**AANKVCGLSTYLKGIAHRV 49

gi|197091019|gb|ACH41863.1| MMKFWFVLLALLGKETHAYY-ENKRNAL**NAT**AANKVCGLSTYLKGIAHRV 49

gi|197091021|gb|ACH41864.1| MMKFWFVLLALLGKETHAYY-ENKRNAL**NAT**AANKVCGLSTYLKGIAHRV 49

gi|161977|gb|AAA30156.1| MMKFWFVLLALLGKETHAYY-ENKRNAL**NAT**AANKVCGLSTYLKGIAHRV 49

gi|197091009|gb|ACH41858.1| MMKFWFVLLALLGKETHAYY-ENKRNAL**NAT**AANKVCGLSTYLKGIAHRV 49

gi|3510713|gb|AAC33573.1| MMKFWFVLLALLGKETHAYY-ENKRNAL**NAT**AANKVCGLSTYLKGIAHRV 49

gi|189094763|emb|CAQ57442.1| -MRFLFVLLALLGKKTHAYY-KNERNAL**NAT**AANKVCALSTYLKGIAHRV 48

gi|189094751|emb|CAQ57428.1| MMRFWFVLLALLGKETYAY--ENERNAL**NAT**AANKVCGLSTYLKGIAHRV 48

gi|197090999|gb|ACH41853.1| -MRFWLVLLALLGKETYANYYENKRNAL**NAT**AANKVCRLSTYLKGIAHRV 49

*:* ::**.:* *: :* :************* ************

gi|197091027|gb|ACH41867.1| NSESAVVTEKLSDLKMRSIQLQLSVMRNRVPSGEKDCKDIRTLLKTVLRN 98

gi|197091011|gb|ACH41859.1| NSESAVVTEKLSDLKMRSIQLQLSVMRNRVPSGEQDCKDIRTLLKTVLRN 98

gi|197090997|gb|ACH41852.1| NSESAVVTEKLSDLKMRSIQLQLSVMRNRVPSGEKDCKDIRTLLKTVLRN 100

gi|197090935|gb|ACH41831.1| NSESAVVTEKLSDLKMRSIQLQLSVMRNRVPSGEKDCKDIRTLLKTVLRN 99

gi|197091003|gb|ACH41855.1| NSESAVVTEKLSDLKMRSIQLQLSVMRNRVPSGEQDCKDIRTLLKTVLRN 98

gi|197091001|gb|ACH41854.1| NSESAVVTEKLSDLKMRSIQLQLSVMRNRVPSGEQDCKDIRTLLKTVLRN 98

gi|197091015|gb|ACH41861.1| NSESAVVTEKLSDLKMRSIQLQLSIMRNRVPSGEKDCKDIRTLLKTVLRN 98

gi|189094728|emb|CAQ57403.1| NSESAVVTEKLSDLKMRSIQLQLSIMRNRVPSGEKDCKDIRTLLKTVLRN 97

gi|197090991|gb|ACH41849.1| NSESAVVTEKLSDLKMRSIQLQLSVMRNRVPSGEKDCKDIRTLLKTVLRN 98

gi|197091023|gb|ACH41865.1| NSESAVVTEKLSDLKMRSIQLQLSVMRNRVPSGEQDCKDIRTLLKTVLRN 97

gi|197091005|gb|ACH41856.1| NSESAVVTEKLSDLKMRSIQLQLSVMRNRVPSGEKDCKDIRTLLKTVLRN 98

gi|189094648|emb|CAQ57310.1| NSESAVVTEKLSDLKMRSIQLQLSVMRNRVPSGEKDCKDIRTLLKTVLRN 97

gi|197091025|gb|ACH41866.1| NGESAVVTEKLSDLKIRSIQLQLSVMRNRVPSGEQDCKDIRTLLKTVLRN 99

gi|197091019|gb|ACH41863.1| NSESAVVTEKLSDLKMRSIQLQLSVMRNRVPSGEQDCKDIRTLLKTVLRN 99

gi|197091021|gb|ACH41864.1| NSESAVVTEKLSDLKMRSIQLQLSVMRNRVPSGEQDCKDIRTLLKTVLRN 99

gi|161977|gb|AAA30156.1| NSESAVVTEKLSDLKMRSIQLQLSVMRNRVPSGEQDCKDIRTLLKTVLRN 99

gi|197091009|gb|ACH41858.1| NSESAVVTEKLSDLKMRSIQLQLSVMRNRVPSGEQDCKDIRTLLKTVLRN 99

gi|3510713|gb|AAC33573.1| NSESAVVTEKLSDLKMRSIQLQLSVMRNRVPSGEQDCKDIRTLLKTVLRN 99

gi|189094763|emb|CAQ57442.1| NSESAVVTEKLSDLKMRSIQLQLSVMRNRVPSGEQDCKDISTLLKTVLRN 98

gi|189094751|emb|CAQ57428.1| NSESAVVTEKLSDLKMRSIQLQLSVMRNRVPSGEKDCKDIRTLLKTVLRN 98

gi|197090999|gb|ACH41853.1| SSESAVVTEKLSDLKMKSIQLQLSILRNRVPSGEKDCKDIRTLLKTVLRN 99

..**.**********::*******::********:***** *:*******

gi|197091027|gb|ACH41867.1| EFTFQQELEEMR**NAS**ALAAAAAGIAAGRLEEWIFVFAQAAGRSSQFCIST 148

gi|197091011|gb|ACH41859.1| EFTFQQELEEMR**NAS**ALAAAAAGLAAGRLEEWIFVFAQAADGSSQFCISV 148

gi|197090997|gb|ACH41852.1| EFTFQQELEEMR**NAS**ALAAAAAGLAAGRLEEWIFVFAQAAGGSSQFCISV 150

gi|197090935|gb|ACH41831.1| EFTFQQELEEMR**NAS**ALAAAAAGLAAGRLEEWIFVFAQAAGGSSQFCISV 149

gi|197091003|gb|ACH41855.1| EFTFQQELEEMR**NAS**ALAAAAAGIAAGRLEEWIFVFAQAADGSSQFCISV 148

gi|197091001|gb|ACH41854.1| EFTFQQELEEMR**NAS**ALAAAAAGLAAGRLEEWIFVFAQAADGSSQFCISV 148

gi|197091015|gb|ACH41861.1| EFTFQQELEEMR**NAS**ALAAAAAGIAAGRLEEWIFVFAQAAGRSSQFCISV 148

gi|189094728|emb|CAQ57403.1| EFTFQQELEEMR**NAS**ALAAAAAGLAAGRLEEWIFVFAQAAGRSSQFCIST 147

gi|197090991|gb|ACH41849.1| EFTFQQELEEMR**N**T**S**ALAAAAAGIAAGRLEEWIFVFAQAAGRSSQFCISV 148

gi|197091023|gb|ACH41865.1| EFTFQQELEEMR**NAS**ALAAAAAGIAAGRLEEWIFVFAQAGGRSSQFCIST 147

gi|197091005|gb|ACH41856.1| EFTFQQELEEMR**N**T**S**ALAAAAAGLAAGRLEEWIFVFAQAADRSSQFCISV 148

gi|189094648|emb|CAQ57310.1| EFTFQQELEEMR**NAS**ALAAAAAGLAAGRLEEWIFVFAQAADRSSQFCISV 147

gi|197091025|gb|ACH41866.1| EFTFQQELEEMR**N**T**S**ALAAAAAGIAAGRLEEWIFVFAQAADRSSQFCISV 149

gi|197091019|gb|ACH41863.1| EFTFQQELEEMR**NAS**ALAAAAAGIAAGRLEEWIFVFAQAAGRSSQFCISV 149

gi|197091021|gb|ACH41864.1| EFTFQQELEEMR**NAS**ALAAAAAGIAAGRLEEWIFVFAQAAGRSSQFCISV 149

gi|161977|gb|AAA30156.1| EFTFQQELEEMR**NAS**ALAAAAAGIAAGRLEEWIFVFAQAAGRSSQFCISV 149

gi|197091009|gb|ACH41858.1| EFTFQQELEEMR**NAS**ALAAAAAGIAAGRLEEWIFVFAQAAGGSSQFCISV 149

gi|3510713|gb|AAC33573.1| EFTFQQELEEMR**NAS**ALAAAAAGIAAGRLEEWIFVFAQAAGGSSQFCISV 149

gi|189094763|emb|CAQ57442.1| EFTFQQELEEMR**NAS**ALAAAAAGLAAGRLEEWIFVFAQAAGRSSQFCISV 148

gi|189094751|emb|CAQ57428.1| EFTFQQELEEMR**NAS**ALAAAAAGLAAGRLEEWIFVFAQAAGGSSQFCISV 148

gi|197090999|gb|ACH41853.1| EFTFQQELEEMR**NAS**ALAAAAAGIAAGRLEEWIFVFAQAAGMTSKFCISV 149

*************:*********:***************.. :*:****.

gi|197091027|gb|ACH41867.1| GKTGPAEYNNLQECFDGTIGPETLYKIEDSRVKESAKTRLLLHEVLSSIS 198

gi|197091011|gb|ACH41859.1| GKTGPAEYNNLQECFDGTIGPETLYKIEDSRVKESAKTRLLLHEVLSSIS 198

gi|197090997|gb|ACH41852.1| GKNIPAEHKNLQECFDGKIGPETLYKIEDSRVKESAQKSLQLHEVLSSIS 200

gi|197090935|gb|ACH41831.1| GRTGPAEYNNLQECFDGKIGPETLYKIEDSRVKESAQKSLQLHEVLSSIS 199

gi|197091003|gb|ACH41855.1| GTNIPAEHNNLQECFDGTIGPETLYKIEDSRVKESAKKSLQLHEALSSIS 198

gi|197091001|gb|ACH41854.1| GTNIPAEHNNLQECFDGTIGPETLYKIEDSRVKESAKKSLQLHEALSSIS 198

gi|197091015|gb|ACH41861.1| GKTGPAEYNNLQECFDGTIGPETLYKIEDSRVKESAKKSLQLHEVLSSIS 198

gi|189094728|emb|CAQ57403.1| GKTGPAEYNNLQECFDGTIGPETLYKIEDSRVKESAKKSLQLHEVLSSIS 197

gi|197090991|gb|ACH41849.1| GKTGPAEYNNLQECFDGTIGPETLYKIEDSRVKESAKTSLQLHEVLSSIS 198

gi|197091023|gb|ACH41865.1| GKTGPAEYNNLQECFDGTIGPETLYKIEDSRVKESAKTSLQLHEVLSSIS 197

gi|197091005|gb|ACH41856.1| GKHIAAEHGNLQECFDGTIGPETLYKIEDSRVKESAKTSLQLHEVLSSIS 198

gi|189094648|emb|CAQ57310.1| GKTIPPEQNNLQECFDGTIGPETLYKIEDSRVKESAKKSLQLHEALSSIS 197

gi|197091025|gb|ACH41866.1| GKTIPPEHNNLQECFDGTIGPETLYKIEDSRVKESAKKSLQLHEALSSIS 199

gi|197091019|gb|ACH41863.1| GKHIPAEHGNLQECFDGIIGPETLYKIEDSRVKESAQKSLQLHEVLSSIS 199

gi|197091021|gb|ACH41864.1| GKHIPAEHGNLQECFDGIIGPETLYKIEDSRVKESAQKSLQLHEVLSSIS 199

gi|161977|gb|AAA30156.1| GKHIPAEHGNLQECFDGIIGPETLYKIEDSRVKESAQKSLQLHEVLSSIS 199

gi|197091009|gb|ACH41858.1| GKHIPAEHGNLQECFDGIIGPETLYKIEDSRVKESAQKSLQLHEVLSSIS 199

gi|3510713|gb|AAC33573.1| GTNIPAEYNNLQECFDGIIGPETLYKIEDSRVKESAQKSLQLHEVLSSIS 199

gi|189094763|emb|CAQ57442.1| GKTIPAEHGDLQECFDGTIGPETLYKIEDSRVKESAKKSLQLHEALSSIS 198

gi|189094751|emb|CAQ57428.1| GKHIAAEHGNLQECFDGKIGPETLYKIEDSRVKESAQKSLQLHEVLSSIS 198

gi|197090999|gb|ACH41853.1| GGSRPAVHDKLQECFDGTIGPETLYKIEDSRVKESAQKSLQLHEALSSIS 199

* .. .******* ******************:. * ***.***:*

gi|197091027|gb|ACH41867.1| FGSLGAENIRGGNGKDGCNLVRTDNNGILKGGSPTRH**NLT**WGGGVMNFGS 248

gi|197091011|gb|ACH41859.1| FGSLGAENIRGGNGKDGCNLVRTDNNGILKGGSPTRH**NLT**WGGGVMNFGS 248

gi|197090997|gb|ACH41852.1| FSSLGAENIRGGNGKDGCNLVRTDNNGILKGGSPTRH**NLT**WGGGVMNFGS 250

gi|197090935|gb|ACH41831.1| FSSLGAENIRGGNGKDGCNLVRTDNNGILKGGSPTRH**NLT**WGGGVMNFGS 249

gi|197091003|gb|ACH41855.1| FSSLGVKNIRGGNGRDGCNLVRTDTNGILNGGSPTRH**NLT**WGGGVMNFGS 248

gi|197091001|gb|ACH41854.1| FSSLGVKNIRGGNGRDGCNLVRTDTNGILNGGSPTRH**NLT**WGGGVMNFGS 248

gi|197091015|gb|ACH41861.1| FGSLGVKNIRGGNGKDRCNLVRTDTDGVLEGGSPTRH**NLT**WGGGVMNFGS 248

gi|189094728|emb|CAQ57403.1| FSSLGVKNIRGGNGKDRCNLVRTDTDGVLEGGSPTRH**NLT**WGGGVMNFGS 247

gi|197090991|gb|ACH41849.1| FGSLGVKNIRGGNGKDGCNLVRTDTDGVLEGGSPTRH**NLT**WGGGVMNFGS 248

gi|197091023|gb|ACH41865.1| FGSLGVKNIRGGNGRDGCNLVRTDTDGVLEGGSPTRH**NLT**WGGGVMNFGS 247

gi|197091005|gb|ACH41856.1| FGSLGVKNIRGGNGRDGCNLVRTDTDGVLEGGSPTRH**NLT**WGGGVMNFGS 248

gi|189094648|emb|CAQ57310.1| FNSLGAESIRGGNGKDGCNLVRTDTDGILNGGSPTRH**NLT**WGGGVMNFGS 247

gi|197091025|gb|ACH41866.1| FGSLGVKNIRGGNGKDGCNLVRTDTDGILNGGSPTRH**NLT**WGGGVMNFGS 249

gi|197091019|gb|ACH41863.1| FNSLGAENIRGGNGRDGCNLVRTDTDGVLEGGSVRRH**NLT**WGGGVMNFGS 249

gi|197091021|gb|ACH41864.1| FNSLGAENIRGGNGRDGCNLVRTDTDGVLEGGSVRRH**NLT**WGGGVMNFGS 249

gi|161977|gb|AAA30156.1| FNSLGAENIRGGNGRHGCNLVRTDTDGVLEGGSVRRH**NLT**WGGGVMNFGS 249

gi|197091009|gb|ACH41858.1| FNSLGAENIRGGNGRHGCNLVRTDTDGVLEGGSVRRH**NLT**WGGGVMNFGS 249

gi|3510713|gb|AAC33573.1| FNSLGAENIRGGNGRDGCNLVRTDTDGVLEGGSVRRH**NLT**WGGGVMNFGS 249

gi|189094763|emb|CAQ57442.1| FSSLGVKNIRGGNGRDGCNLVRTDTNGILEGGSPTRH**NLT**WGGGVMNFGS 248

gi|189094751|emb|CAQ57428.1| FGSLGVKNIRGGNGRDGCNLVRTDTNGILNGGSPTRH**NLT**WGGGVMNFGS 248

gi|197090999|gb|ACH41853.1| FGSLGVKNIRGGNGMDGCNLVRTDTDGILAGGSPTRH**NLT**WGGGVMNFGS 249

*.***.:.****** . *******.:*:* *** ***************

gi|197091027|gb|ACH41867.1| YQ**NGS**MYVEGGEYGDATEYGAVRWTEDPSKVSIFKDVIRLFARFQEAKNA 298

gi|197091011|gb|ACH41859.1| YQ**NGS**MYVEGGEYGDATEYGAVRWTEDPSKVSIFKDVIRLFARFKEAKNA 298

gi|197090997|gb|ACH41852.1| YQ**NGS**MYVEGGEYGDATPHGTVRWTEDPNKVSIFKDVIRLFARFKEAKNA 300

gi|197090935|gb|ACH41831.1| YQ**NGS**MYVEGGEYGDATPHGTVRWTEDPNKVSIFKDVIRLFARFKEAKNA 299

gi|197091003|gb|ACH41855.1| YQ**NGS**MYVEGGEYGDATEYGAVRWTEDPSKVSIFEDLIRLFARFQEAKNA 298

gi|197091001|gb|ACH41854.1| YQ**NGS**MYVEGGEYGDATEYGAVRWTEDPSKVSIFKDVIRLFARFQEAKNA 298

gi|197091015|gb|ACH41861.1| YQ**NGS**MYVEGGEYGDATEYGAVRWTEDPSKVSIFKDVIRLFARFQEAKNA 298

gi|189094728|emb|CAQ57403.1| YQ**NGS**MYVEGGEYGDATEYGAVRWTEDPSKVSIFKDVIRLFARFQEAKNA 297

gi|197090991|gb|ACH41849.1| YQ**NGS**MYVEGGEYGDATEYGAVRWTDDPSKVSIFKDVIRLFARFQEAKNA 298

gi|197091023|gb|ACH41865.1| YQ**NGS**MYVEGGEYGDATEYGAVRWTEDPSKVSIFKDVIRLFARFQEAKNA 297

gi|197091005|gb|ACH41856.1| YQ**NGS**MYVEGGEYGDATEYGAVRWTEDPSKVSIFKDVIRLFARFQEAKNA 298

gi|189094648|emb|CAQ57310.1| YQ**NGS**MYVEGGEYGDATEYGAVRWTEDPSKVSIFKDVIRLFARFQEAKNA 297

gi|197091025|gb|ACH41866.1| YQ**NGS**MYVEGGEYGDATEYGAVRWTEDPSKVSIFKDVIRLFARFQEAKNA 299

gi|197091019|gb|ACH41863.1| YQ**NGS**MYVEGGEYGDATEYGAVRWTEDPSKVSIFKDVIRLFARFQEAKNE 299

gi|197091021|gb|ACH41864.1| YQ**NGS**MYVEGGEYGDATEYGAVRWTEDPSKVSIFKDVIRLFARFQEAKNE 299

gi|161977|gb|AAA30156.1| YQ**NGS**MYVEGGEYGDATEYGAVRWTEDPSKVSIFKDVIRLFARFQEAKNE 299

gi|197091009|gb|ACH41858.1| YQ**NGS**MYVEGGEYGDATEYGAVRWTEDPSKVSIFKDVIRLFARFQEAKNE 299

gi|3510713|gb|AAC33573.1| YQ**NGS**MYVEGGEYGDATEYGAVRWTEDPSKVSIFKDVIRLFARFQEAKNE 299

gi|189094763|emb|CAQ57442.1| YQ**NGS**MYVEGGEYGDATEYGAVRWTEDPSKVSIFEDVIRLFARFQEAKNE 298

gi|189094751|emb|CAQ57428.1| YQ**NGS**MYVEGGEYGDATEYGAVRWTEDPSKVSIFEDVIRLFARFQEAKNA 298

gi|197090999|gb|ACH41853.1| YQ**NGS**MYVEGGEYGDATEYGAVRWTKDPSKVSIFKDVIRLFARFQEAKNA 299

***************** :*:****.**.*****:*:*******:****

gi|197091027|gb|ACH41867.1| VMKKIKTTVDELTKCIGQKEAELTNDQIYEEFIWETINRLELSKRVSEQP 348

gi|197091011|gb|ACH41859.1| VMTKIKTTVDELTKCIGQKEAELTNDQIYEEFIWETINRLELSKRVSEQP 348

gi|197090997|gb|ACH41852.1| VMTKIKTTVDELTKCIGQKEAELTNDQLYEEFIWETINRLELSKRVSEQP 350

gi|197090935|gb|ACH41831.1| VMTKIKTTVDELTKCIGQKEAELTNDQLYEEFIWETINRLELSKRVSEQP 349

gi|197091003|gb|ACH41855.1| VMKKIKTTVDELTKCIGQKEAELTNDQIYEEFIWETINRLELSKRMSEQS 348

gi|197091001|gb|ACH41854.1| VMRRIKTTVDELTKCIGQKEAELTNDQIYEEFIWETINRLELSKRMSEQP 348

gi|197091015|gb|ACH41861.1| VMKKIKTTVDELTKCIGQKEAELTNDQLYEEFIWETINRLELSKRVSEQS 348

gi|189094728|emb|CAQ57403.1| VMKKIKTTVDELTKCIGQKEAELTNDQLYEEFIWETINRLELSKRVSEQS 347

gi|197090991|gb|ACH41849.1| VMKKIKTTVDELTKCVGQKEAELTNDQLYEEFIWETINRLELSKRVSEQS 348

gi|197091023|gb|ACH41865.1| VMTKIKTTVDELTKCIGHKEAELTNDQLYEEFIWETINRLELSKRVSEQS 347

gi|197091005|gb|ACH41856.1| VMTKIKTTVDELTKCIGQKEAELTNDQLYEEFIWETINRLELSKRVSEQS 348

gi|189094648|emb|CAQ57310.1| VMKKIKTTVDELTKCIGQKEAELTNDQLYEEFIWETINRLELSKRVSEQS 347

gi|197091025|gb|ACH41866.1| VMKKIKTTVDELTKCTGQKEAELTNDQLYEEFIWETINRLELSKRVSEQS 349

gi|197091019|gb|ACH41863.1| VMNKIKTTVDELAKCIGQKEVELTDDQLYEEFIWETIHRLELSKRVSEQL 349

gi|197091021|gb|ACH41864.1| VMNKIKTTVDELAKCIGQKEVELTDDQLYEEFIWETIHRLELSKRVSEQL 349

gi|161977|gb|AAA30156.1| VMNKIKTTVDELAKCIGQKEVELTDDQLYEEFIWETIHRLELSKRVSEQL 349

gi|197091009|gb|ACH41858.1| VMNKIKTTVDELAKCIGQKEVELTDDQLYEEFIWETIHRLELSKRVSEQL 349

gi|3510713|gb|AAC33573.1| VMNKIKTTVDELAKCIGQKEVELTDDQLYEEFIWETIHRLELSKRVSEQL 349

gi|189094763|emb|CAQ57442.1| VMNKIKTTVDELAKCIGQKEVELTDDQLYEEFIWETIHRLELSKRVSEQP 348

gi|189094751|emb|CAQ57428.1| VMRRIKTTVDELTKCIGQKEAELTNDQIYEEFIWETIHRLELSKRVSEQP 348

gi|197090999|gb|ACH41853.1| VMNKIKTTVDELTKCIGHKEAELTDYQLYEEFIWETIHRLELSKRVSEQP 349

** :**:*****:** *:**.***: *:***

gi|197091027|gb|ACH41867.1| SLGEEEETILKS**NYT**AEPVRGPFTGAGANTVAVQSSVFTAALCCSALLLG 398

gi|197091011|gb|ACH41859.1| SLGEEEETILKS**NYT**AEPVRGPFTGAGANTVAVQSSVSTAALCCSVLLLG 398

gi|197090997|gb|ACH41852.1| SLGEEEETILKS**NYT**AEPVRGPFTGAGANTVAVQSSVSTAALCCSVLLFG 400

gi|197090935|gb|ACH41831.1| SLGEEEETILKS**NYT**AEPVRGPFTGAGANTVAVQSSVSTAALCCSVLLFG 399

gi|197091003|gb|ACH41855.1| AFGEEEETILKS**NYT**AEPVRGPFTGAGSNTVAVHLSFSTAALCCSVLLLG 398

gi|197091001|gb|ACH41854.1| TLGEEEETILKS**NYT**AEPVRGPFTGAGANTVALHLSVSTAALCCSVLLLG 398

gi|197091015|gb|ACH41861.1| AFGEEEETIVKF**NYT**AEPVRGPFTVAGANAAAIHLSVSTAALCRSALLLG 398

gi|189094728|emb|CAQ57403.1| AFGEEEETIVKF**NYT**AEPVRGPFTVAGANAAAIHLSVSTAALCRSALLLG 397

gi|197090991|gb|ACH41849.1| AFGEEEETIVKF**NYT**AEPVRGPFTVAGANAAAIHLSVSTAALCRSALLLG 398

gi|197091023|gb|ACH41865.1| AFGEEEETIVKF**NYT**AEPVRGPFTVAGANAAAIHLSVSTAALCRSALLLG 397

gi|197091005|gb|ACH41856.1| AFGEEEETIVKF**NYT**AEPVRGPFTVAGANAAAIHLSVSTAALCRSALLLG 398

gi|189094648|emb|CAQ57310.1| AFGEEEETIVKF**NYT**AEPVRGPFTVAGANAAAIHLSVSTAALCRSALLLG 397

gi|197091025|gb|ACH41866.1| AFGEEEETILKS**NYT**AEPVRGPFTVAGSNAVAIHLSVSTAALCRSALLLG 399

gi|197091019|gb|ACH41863.1| SLGEEEETILKS**NYT**AEPVRGPFTVAGSNAAAVHLSVSTAALCFSVLLLG 399

gi|197091021|gb|ACH41864.1| SLGEEEETILKS**NYT**AEPVRGHFTVAGSNAAAVHLSVSTAALCFSVLLLG 399

gi|161977|gb|AAA30156.1| SLGEEEETILKS**NYT**AEPVRGPFTVAGSNAAAVHLSVSTAALCFSVLLLG 399

gi|197091009|gb|ACH41858.1| SLGEEEETILKS**NYT**AEPVRGPFTVAGSNAAAVHLSVSTAALCFSVLLLG 399

gi|3510713|gb|AAC33573.1| SLGEEEETILKS**NYT**AEPVRGPFTVAGSNTVAVHLSVSTAALCFSVLLLG 399

gi|189094763|emb|CAQ57442.1| SLGEEEETILKS**NYT**AEPVRGPFTGAGSNTVAVHLSVSTAALCRSALLLG 398

gi|189094751|emb|CAQ57428.1| SLGEEEETILKS**NYT**AEPVRGPFTGAGSNTVAVHLSVSTAALCCLALLLG 398

gi|197090999|gb|ACH41853.1| SLGEQEETILKS**NYT**AEPVRGPFTGAGADAAAIQSSVSTAALCCLALLLG 399

gi|197091027|gb|ACH41867.1| VL 400

gi|197091011|gb|ACH41859.1| VL 400

gi|197090997|gb|ACH41852.1| VL 402

gi|197090935|gb|ACH41831.1| VL 401

gi|197091003|gb|ACH41855.1| VL 400

gi|197091001|gb|ACH41854.1| VL 400

gi|197091015|gb|ACH41861.1| VL 400

gi|189094728|emb|CAQ57403.1| VL 399

gi|197090991|gb|ACH41849.1| VL 400

gi|197091023|gb|ACH41865.1| VL 399

gi|197091005|gb|ACH41856.1| VL 400

gi|189094648|emb|CAQ57310.1| VL 399

gi|197091025|gb|ACH41866.1| VL 401

gi|197091019|gb|ACH41863.1| VL 401

gi|197091021|gb|ACH41864.1| VL 401

gi|161977|gb|AAA30156.1| VL 401

gi|197091009|gb|ACH41858.1| VL 401

gi|3510713|gb|AAC33573.1| VL 401

gi|189094763|emb|CAQ57442.1| VL 400

gi|189094751|emb|CAQ57428.1| VL 400

gi|197090999|gb|ACH41853.1| VM 401
