# Supplementary material for: Modeling of the N-Glycosylated Transferrin Receptor Suggests How Transferrin Binding Can Occur within the Surface Coat of Trypanosoma brucei
Source: PLoS Pathog. 2012 Apr 5;8(4):e1002618. doi: 10.1371/journal.ppat.1002618 (PMC3320590; doi:10.1371/journal.ppat.1002618)
Supplement: Table S2 — CLUSTAL 2.1 multiple sequence alignment of 14 T. brucei brucei ESAG7 sequences. The sequences in general and the N-glycosylation sites (in bold) in particular are highly conserved in the different ESAG6 family members. (DOC) [file ppat.1002618.s005.doc]

**Table S2. CLUSTAL 2.1 multiple sequence alignment of 14 T. brucei brucei ESAG7 sequences.**

The sequences in general and the N-glycosylation sites (in bold) in particular are highly conserved in the different ESAG6 family members.

gi|189094762|emb|CAQ57441.1| -MRFLFVLLALLGKKTHAYYK-NERNAL**NAT**AANKVCALSTYLKGIAHRV 48

gi|189094771|emb|CAQ57452.1| -MRFWFVLLALLGKETHAYYYENKRNAL**NAT**AANKVCALSTYLKGIAHRV 49

gi|189094752|emb|CAQ57429.1| MMRFWFVLLALLGKKTHAYYE-NERNAL**NAT**AANKVCGLSTYLKGVAHRV 49

gi|189094727|emb|CAQ57402.1| -MRFWFVLLALLGKEIYAYEN--ERNAL**NAT**AANKVCGLSTYLKGIAHRV 47

gi|189094783|emb|CAQ57467.1| -MRFWFVLLALLGKEIYAYEN--ERNAL**NAT**AANKVCGLSTYLKGIAHRV 47

gi|189094744|emb|CAQ57420.1| MMRFWFVLLALLGKETYAYEN--ERNAL**NAT**AANKVCGLSTYLKGIAHRV 48

gi|189094647|emb|CAQ57309.1| -MRFWFVLLALLGKEIYAYEN--ERNAL**NAT**AANKVCGLSTYLKGIAHRV 47

gi|189094723|emb|CAQ57399.1| -MRFWFVLLALLGKETYAYYE-NKRNAL**NAT**AANKVCGLSTYLKGIAHRV 48

gi|189094666|emb|CAQ57332.1| MMKFWFVLLALLGKETHANYYENERNAL**NAT**AANKVCGLSTYLKGIAHRV 50

gi|189094677|emb|CAQ57345.1| MMKFWFVLLALLGKETHANYYENERNAL**NAT**AANKVCGLSTYLKGIAHRV 50

gi|189094616|emb|CAQ57274.1| MMKFWFVLLALLGKETHA-YYENKRNAL**NAT**AANKVCGLSTYLKGIAHRV 49

gi|189094690|emb|CAQ57360.1| MMRFLFVLLALLGKETHA-YYENKRNAL**NAT**AANKVCGLSTYLKGIAHRV 49

gi|161976|gb|AAA30155.1| MMKFWFVLLALLGKETHA-YYENKRNAL**NAT**AANKVCGLSTYLKGIAHRV 49

gi|189094732|emb|CAQ57407.1| MMRFWFVLLALLGKETYA-YYENERNAL**NAT**AANKVCGLSTYLKGIAHRV 49

*:* *********: :* :*************.*******:****

gi|189094762|emb|CAQ57441.1| NSESAVVTEKLSDLKMRSIQLQLSVMRNRVPSGEQDCKDISTLLKTVLRN 98

gi|189094771|emb|CAQ57452.1| NSESAVVTEKISDLKMRSIQLQLSIMRNRVPSGEKDCKDIRTLLKTVLRN 99

gi|189094752|emb|CAQ57429.1| NSESAVVTEKLSDLKMRSIQLQLSVMRNRVPSGEKDCKDIRTLLKTVLRN 99

gi|189094727|emb|CAQ57402.1| NSESAVVTEKLSDLKMRSIQLQLSIMRNRVPSGEKDCKDIRTLLKTVLRN 97

gi|189094783|emb|CAQ57467.1| NSESAVVTEKLSDLKMRSIQLQLSIMRNRVPSGEKDCKDIRTLLKTVLRN 97

gi|189094744|emb|CAQ57420.1| NSESAVVTEKLSDLKMRSIQLQLSVMRNRVPSGEKDCKDIRTLLKTVLRN 98

gi|189094647|emb|CAQ57309.1| NSESAVVTEKLSDLKMRSIQLQLSVMRNRVPSGEKDCKDIRTLLKTVLRN 97

gi|189094723|emb|CAQ57399.1| NSESAVVTEKLSDLKMRSVQLQLSVMRNRVPSGEKDCKDIRTLLKTVLRN 98

gi|189094666|emb|CAQ57332.1| NSESAVVTEKLSDLKMRSIQLQLSVMRNRVPSGEQDCKDIRTLLKTVLRN 100

gi|189094677|emb|CAQ57345.1| NSESAVVTEKLSDLKMRSIQLQLSVMRNRVPSGEQDCKDIRTLLKTVLRN 100

gi|189094616|emb|CAQ57274.1| NSESAVVTEKLSDLKMRSIQLQLSVMRNRVPSGEQDCKDIRTLLKTVLRN 99

gi|189094690|emb|CAQ57360.1| NSESAVVTEKLSDLKMRSIQLQLSVMRNRVPSGEQDCKDIRTLLKTVLRN 99

gi|161976|gb|AAA30155.1| NSESAVVTEKLSDLKMRSIQLQLSVMRNRVPSGEQDCKDIRTLLKTVLRN 99

gi|189094732|emb|CAQ57407.1| NSESAVVTEKLSDLKMRSIQLQLSVMRNRVPSGEKDCKDIRTLLKTVLRN 99

**********:*******:*****:*********:***** *********

gi|189094762|emb|CAQ57441.1| EFTFQQELEEMR**NAS**ALAAAAAGLAAGRLEEWIFVFAQAADRSSQFCISV 148

gi|189094771|emb|CAQ57452.1| EFTFQQELEEMR**NAS**ALAAAAAGIAAGRLEEWIFVFAQAAGRSSQFCISV 149

gi|189094752|emb|CAQ57429.1| EFTFQQELEEMR**NAS**ALAAAAAGLAAGRLEEWIFVFAQAADRSSQFCISV 149

gi|189094727|emb|CAQ57402.1| EFTFQQELEEMR**NAS**ALAAAAAGIAAGRLEEWIFVFAQAADRSSQFCISV 147

gi|189094783|emb|CAQ57467.1| EFTFQQELEEMR**NAS**ALAAAAAGIAAGRLEEWIFVFAQAAGMSSQFCISV 147

gi|189094744|emb|CAQ57420.1| EFTFQQELEEMR**NAS**ALAAAAAGLAAGRLEEWIFVFAQAADRSSQFCISV 148

gi|189094647|emb|CAQ57309.1| EFTFQQELEEMR**NAS**ALAAAAAGIAAGRLEEWIFVFAQAADGSSQFCISV 147

gi|189094723|emb|CAQ57399.1| EFTFQQELEEMR**N**E**S**ALAAAAAGIAAGRLEEWIFVFAQAADGSSQFCISV 148

gi|189094666|emb|CAQ57332.1| EFTFQQELEEMR**NAS**ALAAAAAGIAAGRLEEWIFVFAQAAGGSSQFCISV 150

gi|189094677|emb|CAQ57345.1| EFTFQQELEEMR**NAS**ALAAAAAGIAAGRLEEWIFVFAQAAGGSSQFCISV 150

gi|189094616|emb|CAQ57274.1| EFTFQQELEEMR**NAS**ALAAAAAGIAAGRLEEWIFVFAQAAGGSSQFCISV 149

gi|189094690|emb|CAQ57360.1| EFTFQQELKEMR**NAS**ALAAAAAGIAAGRLEEWIFVFAQAAGGSSQFCISV 149

gi|161976|gb|AAA30155.1| EFTFQQELEEMR**NAS**ALAAAAAGIAAGRLEEWIFVFAQAAGGSSQFCISV 149

gi|189094732|emb|CAQ57407.1| EFTFQQELEEMR**NAS**ALAAAAAGIAAGRLEEWIFVFAQAADMTSQFCISV 149

********:**** *********:****************. :*******

gi|189094762|emb|CAQ57441.1| GKHIAAEHGDLQECFDGTIGPETLYKIEDSRVKESAKKSLQLHEALSSIS 198

gi|189094771|emb|CAQ57452.1| GKTIPAEHGDLQECFDGTIGPETLYKIEDSRVKESAKKSLQLHEALSSIS 199

gi|189094752|emb|CAQ57429.1| GKHIAAEHGNLQECFDGTIGPETLYKIEDSRVKESAQKSLQLHEALSSIS 199

gi|189094727|emb|CAQ57402.1| GKHIAAEHGNLQECFDGTIGPETLYKIEDSRVKESAKKSLQLHEALSSIS 197

gi|189094783|emb|CAQ57467.1| GKTIPAEHGDLQECFDGTIGPETLYKIEDSRVKESAKKSLQLHEALSSIS 197

gi|189094744|emb|CAQ57420.1| GKTIPAEHGDLQECFDGKIGPETLYKIEDSRVKESAKKSLQLHEALSSIS 198

gi|189094647|emb|CAQ57309.1| GKHIPPEHKNLQECFDGTIGPETLYKIEDSRVKESAKKSLQLHEALSSIS 197

gi|189094723|emb|CAQ57399.1| GKHIPAEHGNLQECFDGTIGPETLYKIEDSRVKESAKKSLQLHEALSSIS 198

gi|189094666|emb|CAQ57332.1| GTNIPAEYNNLQECFDGTIGPETLYKIEDSRVKESAQKSLQLHEVLSSIS 200

gi|189094677|emb|CAQ57345.1| GTNIPAEYNNLQECFDGTIGPETLYKIEDSRVKESAQKSLQLHEVLSSIS 200

gi|189094616|emb|CAQ57274.1| GTNIPAEYNNLQECFDGIIGPETLYKIEDSRVKESAQKSLQLHEVLSSIS 199

gi|189094690|emb|CAQ57360.1| GTNIPAEYNNLQECFDGTIGPETLYKIEDSRVKESAQKSLQLHEVLSSIS 199

gi|161976|gb|AAA30155.1| GKHIPAEHGNLQECFDGIIGPETLYKIEDSRVKESAQKSLQLHEVLSSIS 199

gi|189094732|emb|CAQ57407.1| GKNIPAEHKNLQECFNGKIGPETLYKIEDSRVKESAQKSLQLHEVLSSIS 199

*. *..*: :*****:* ******************:*******.*****

gi|189094762|emb|CAQ57441.1| FSSLGAENIVEKGENRGCNLMRTADEGLLKDVCLNR**NFT**WGGGVLNFGYC 248

gi|189094771|emb|CAQ57452.1| FSSLGAENIVEKGENRGCNLMRTADEGLLKDVCLNR**NFT**WGGGVMNFGYC 249

gi|189094752|emb|CAQ57429.1| FSSLGAENIVEKGENRGCNLMRTAYGGLLEGICLNR**NFT**WGGGVMNFGSC 249

gi|189094727|emb|CAQ57402.1| FSSLGAESIIERNEDRGCNLMRTADGGLLKDVCLNR**NFT**WGGGVLNFGYC 247

gi|189094783|emb|CAQ57467.1| FSSLGAESIVEKGENRGCNLMRTADGGLLKDVCLNR**NFT**WGGGVLNFGYC 247

gi|189094744|emb|CAQ57420.1| FSSLGAENIVEQRKNRGCNLMRTAYGGLLKDVCLNR**NFT**WGGGVMNFRSC 248

gi|189094647|emb|CAQ57309.1| FSSLGAESIVEQRKNRGCNLMRTAYGGLLKDFCLNR**NFT**WGGGVMNFGSC 247

gi|189094723|emb|CAQ57399.1| FNSLGAESIVEQGENRGCNLMRTADGGLLKDICLNR**NFT**WGGGVLNFGYC 248

gi|189094666|emb|CAQ57332.1| FSSLGAESIVEQRKNRGCNLMRTADGGLLKDICLNC**NFT**WGGGVMNFGSC 250

gi|189094677|emb|CAQ57345.1| FSSLGAESIVEQRKNRGCNLMRTADGGLLKDICLNC**NFT**WGGGVMNFGSC 250

gi|189094616|emb|CAQ57274.1| FSSLGAESIVEQRKNRGCNLMRTADGGLLKDICLNC**NFT**WGGGVMNFGSC 249

gi|189094690|emb|CAQ57360.1| FSSLGAESIVEQRKNRGCNLMRTADGGLLKDICLNC**NFT**WGGGVMNFGSC 249

gi|161976|gb|AAA30155.1| FSSLGAESIVEQGENRGCNLMRTADGGLLKDICLNC**NFT**WGGGVMNFGSC 249

gi|189094732|emb|CAQ57407.1| FSSLGAESIVERRENRGCNLMRTGRGGLLKDVCLNR**NFT**WGGGVMNFGSC 249

*.*****.*:*: ::********. ***:..*** ********:** *

gi|189094762|emb|CAQ57441.1| VAGNLKIKGGEYGDVGSHDAVRWTEDPSKVSIFKDVIRLFARFQEAKNEV 298

gi|189094771|emb|CAQ57452.1| VAGNLKIKGGEYGDVSSHDAVRWTEDPSKVSIFKDVIRLFARFQEAKNAV 299

gi|189094752|emb|CAQ57429.1| VAGNLEIKGGEYGDVSSHDAVRWTEDPSKVSIFKDVIRLFARFQEAKNAV 299

gi|189094727|emb|CAQ57402.1| VAGNLKIKGGEYGDVGSHDAVRWTEDPSKVSIFKDVIRLFARFQEAKNAV 297

gi|189094783|emb|CAQ57467.1| VAGNLKIKGGEYGDVGSHDAVRWTEDPSKVSIFKDVIRLFARFQEVKNAV 297

gi|189094744|emb|CAQ57420.1| VAGNLKIEGGEYGDVGSHDAVRWTEDPSKVSIFKDVIRLFARFQEAKNAV 298

gi|189094647|emb|CAQ57309.1| VAGNLKIEGGEYGDVGSHDAVRWTEDPSKVSIFKDVIRLFARFQEVKNAV 297

gi|189094723|emb|CAQ57399.1| VAGNLKIKGGEYGDVSSHDAVRWAEDPNKVSIFKDVIRLFARFQEAKNAV 298

gi|189094666|emb|CAQ57332.1| VAGNLKIKGGEYGDVSSHDVVRWTEDPSKVSIFKDVIRLFARFQEAKNAV 300

gi|189094677|emb|CAQ57345.1| VAGNLKIKGGEYGDVSSHDVVRWTEDPSKVSIFKDVIRLFARFEEAKNAV 300

gi|189094616|emb|CAQ57274.1| VAGNLKIKGGEYGDVSSHDVVRWTEDPSKVSIFKDVIRLFARFQEAKNAV 299

gi|189094690|emb|CAQ57360.1| VAGNLKIKGGEYGDVSSHDVVRWTEDPSKVSIFKDVIRLFARFQEAKNAV 299

gi|161976|gb|AAA30155.1| VAGNLKIKGGEYGDVSSHDVVRWTEDPSKVSIFKDVIRLFARFQEAKNAV 299

gi|189094732|emb|CAQ57407.1| VAGNLKIKGGEYDDVSSHDEVRWTEDPSKVSIFKDVIRLFARFKEAKNAV 299

*****:*:****.**.*** ***:***.***************:*.** *

gi|189094762|emb|CAQ57441.1| MNKIKTTVDELAKCIGQKEVELTDDQLYEEFEAIQKYLGFL--- 339

gi|189094771|emb|CAQ57452.1| MTKIKTTVDELTKCIGQKEAELTNDQIYEEFEAIQKYLGFL--- 340

gi|189094752|emb|CAQ57429.1| MRRIKTTVDELTKCIGKKEAELTNDQIYEEFEAIQKYLGFL--- 340

gi|189094727|emb|CAQ57402.1| MKKIKTTVDELTKCIGQKEAELTNDQIYEEFEAIQKYLGFL--- 338

gi|189094783|emb|CAQ57467.1| VKKIKTTVDELTKCIGQKEAELTNDQLYEEFEVIQKYLWFL--- 338

gi|189094744|emb|CAQ57420.1| MKKIKTTVDELTKCIGQKEAELTNDQLYEEFEAIQKYLGFL--- 339

gi|189094647|emb|CAQ57309.1| MKKIKTTVDELTKCIGQKEAELTNDQLYEEFEAIQKYLWFL--- 338

gi|189094723|emb|CAQ57399.1| MKKIKSTVDELTKCIGKKEAELTNDQLYEEFEAIQKYLGSL--- 339

gi|189094666|emb|CAQ57332.1| MNKIKTTVDELAKCIGQKEVELTNDQLYEEFEAIQKYLGSL--- 341

gi|189094677|emb|CAQ57345.1| MNKIKTTVDELAKCIGQKEVELTNDQLYEEFEAIQKYLGSL--- 341

gi|189094616|emb|CAQ57274.1| MNKIKTTVDELAKCIGQKEVELTNDQLYEEFEAIQKYLGSL--- 340

gi|189094690|emb|CAQ57360.1| MNKIKTTVDELAKCIGQKEVELTNDQLYEEFEAIQKYLGSL--- 340

gi|161976|gb|AAA30155.1| MNKIKTTVDELAKCIGQKEVELTNDQLYEEFEAIQKYLGSL--- 340

gi|189094732|emb|CAQ57407.1| MKKIKTTVDELTKCIGQKEAELTNDQLYEEFEAIQKYLGFGKME 343

: :**:*****:****:**.***:**:*****.*****
